# Drought soil legacy overrides maternal effects on plant growth

**DOI:** 10.1111/1365-2435.13341

**Published:** 2019-04-29

**Authors:** Jonathan R. De Long, Marina Semchenko, William J. Pritchard, Irene Cordero, Ellen L. Fry, Benjamin G. Jackson, Ksenia Kurnosova, Nicholas J. Ostle, David Johnson, Elizabeth M. Baggs, Richard D. Bardgett

**Affiliations:** ^1^ School of Earth and Environmental Sciences The University of Manchester Manchester UK; ^2^ Department of Terrestrial Ecology Netherlands Institute of Ecology Wageningen The Netherlands; ^3^ The Global Academy of Agriculture and Food Security, The Royal (Dick) School of Veterinary Studies The University of Edinburgh Midlothian UK; ^4^ Lancaster Environment Centre Lancaster University Lancaster UK

**Keywords:** ^15^N, above‐ground–below‐ground interactions, climate change, climate extremes, drought shelters, extracellular soil enzymes, mycorrhizae, plant diversity

## Abstract

1. Maternal effects (i.e. trans‐generational plasticity) and soil legacies generated by drought and plant diversity can affect plant performance and alter nutrient cycling and plant community dynamics. However, the relative importance and combined effects of these factors on plant growth dynamics remain poorly understood.
2. We used soil and seeds from an existing plant diversity and drought manipulation field experiment in temperate grassland to test maternal, soil drought and diversity legacy effects, and their interactions, on offspring plant performance of two grassland species (*Alopecurus pratensis* and *Holcus lanatus*) under contrasting glasshouse conditions.
3. Our results showed that drought soil legacy effects eclipsed maternal effects on plant biomass. Drought soil legacy effects were attributed to changes in both abiotic (i.e. nutrient availability) and biotic soil properties (i.e. microbial carbon and enzyme activity), as well as plant root and shoot atom ^15^N excess. Further, plant tissue nutrient concentrations and soil microbial C:N responses to drought legacies varied between the two plant species and soils from high and low plant diversity treatments. However, these diversity effects did not affect plant root or shoot biomass.
4. These findings demonstrate that while maternal effects resulting from drought occur in grasslands, their impacts on plant performance are likely minor relative to drought legacy effects on soil abiotic and biotic properties. This suggests that soil drought legacy effects could become increasingly important drivers of plant community dynamics and ecosystem functioning as extreme weather events become more frequent and intense with climate change.

A plain language summary is available for this article.

## 1 INTRODUCTION

Plant performance can be directly affected by several environmental factors, including temperature, precipitation and nutrient availability. However, in the long term, indirect effects manifested through soil legacy (De Long, Kardol, Sundqvist, Veen, & Wardle, 2015; Kaisermann, de Vries, Griffiths, & Bardgett, 2017) or maternal effects (Mousseau, Uller, Wapstra, & Badyaev, 2009; Verhoeven & van Gurp, 2012; Walter, Harter, Beierkuhnlein, & Jentsch, 2016) resulting from environmental change could also impact negatively or positively on plant performance. Further, interactions between soil legacy and maternal effects could result in synergistic, additive or cancellation effects on plant performance. However, little is known about how soil legacy and maternal effects individually and interactively affect plant growth and soil processes.

Changes in environmental conditions can alter soil abiotic and biotic properties, thereby generating soil legacy effects that alter plant performance. For example, higher temperatures can increase microbial activity, potentially increasing soil nutrient availability. This can create temperature legacy effects on soil that increase plant growth even when plants are grown under lower temperatures (De Long et al., 2015). The legacy effects of drought can lead to nutrient pulses once soils are rewetted and cause lasting changes in soil microbial communities (Birch, 1958; Bloor & Bardgett, 2012; Evans & Wallenstein, 2012; Leitner et al., 2017). These changes can lead to altered plant–soil feedbacks and plant competitive interactions (Kaisermann et al., 2017). Drought‐induced shifts in the relative abundance of fungi and bacteria can be mitigated by subordinate species, with implications for ecosystem functioning (Mariotte, Robroek, Jassey, & Buttler, 2015). As a result, soil legacy effects could affect plant species dominance and ecosystem functioning.

Plants can change the fitness of their offspring through maternal effects. Such effects occur when a maternal plant growing under certain environmental conditions produces offspring that are better or worse adapted to cope with the conditions experienced by their mother (Herman & Sultan, 2011; Roach & Wulff, 1987). Essentially, plants can influence which genes are switched “on” or “off” in their offspring (i.e. trans‐generational epigenetics) or alter resource provisioning to their offspring. This allows for the trans‐generational conveyance of information or resources (Shea, Pen, & Uller, 2011). Maternal effects are favoured when environmental cues in the maternal generation are correlated with selecting factors in the offspring generation (Burgess & Marshall, 2014). Environmental stressors can result in both adaptive and maladaptive maternal effects. For example, maternal plants of *Aegilops triuncialis*, an invasive grass, grown under drought and nutrient‐limited conditions conveyed greater stress tolerance to their offspring via increased photosynthetic efficiency (Dyer et al., 2010). Conversely, maternal *Persicaria hydropiper* plants grown under drought conditions produced seedlings that performed worse under both ambient and drought conditions (Sultan, Barton, & Wilczek, 2009). Maternal effects resulting from extreme rain events can change leaf stoichiometry and improve growth rates (Walter et al., 2016). Maternal effects can be cumulative over multiple generations, with parental and grandparental drought stress resulting in seedlings better adapted to cope with subsequent drought (Herman, Sultan, Horgan‐Kobelski, & Riggs, 2012). The dominance of a plant species might be jeopardized if environmental stressors (e.g. increased temperature) lead to maladaptive maternal effects (Hovenden et al., 2008). Although our understanding of maternal effects in plants is increasing, links between maternal effects and extreme climate events remain poorly understood.

Importantly, drought soil legacies and maternal effects can interact with plant community diversity. Plant species diversity can affect soil microbial community composition and function (De Deyn, Quirk, & Bardgett, 2011; Metcalfe, Fisher, & Wardle, 2011), which can buffer grassland productivity against the deleterious effects of drought (Craven et al., 2016). Alternatively, direct negative effects of drought on soil microbes could override the ability of high plant diversity to buffer against drought (Vogel, Eisenhauer, Weigelt, & Scherer‐Lorenzen, 2013). Further, the legacy of plant community diversity can create maternal effects, with higher diversity resulting in reduced growth and reproduction in the next generation (Rottstock, Kummer, Fischer, & Joshi, 2017), potentially leading to shifts in species' dominance. In line with this, seedlings from high diversity maternal origin may perform worse when grown on low diversity legacy soils (Zuppinger‐Dingley, Flynn, De Deyn, Petermann, & Schmid, 2016). Further, drought legacies can lead to decreased competitive ability of dominant plants and possible post‐drought competitive release for subordinate species, with implications for plant community diversity (Mariotte, Vandenberghe, Kardol, Hagedorn, & Buttler, 2013). Therefore, interactions between plant diversity, drought soil legacies and maternal effects could have implications for the maintenance of species dominance. Despite these advances, our knowledge is limited as to how plant diversity legacy effects interact with drought soil legacy and maternal effects.

Our aim was to examine the relative and interactive importance of maternal effects and soil legacy of drought and plant diversity on plant performance in temperate grassland. We conducted a glasshouse experiment using a dominant (*Holcus lanatus* L.) and a subordinate (*Alopecurus pratensis* L.) grass species, and soils and seeds that were collected from an established field experiment in which plant community composition was manipulated (Leff et al., 2018). We tested the following predictions: (a) plants grown from seeds of drought origin will perform better than those from seeds of ambient (i.e. non‐drought) origin when grown under drought conditions due to maternal effects that make them better suited to cope with drought; (b) plants will experience strong drought soil legacy effects that will interact with maternal effects in context‐dependent ways; (c) high plant community diversity will dampen both maternal and soil legacy effects of drought (i.e. interactions between community diversity and maternal environment or drought legacy); and (d) maternal effects and responses to soil legacies will differ between dominant and subordinate species (i.e. interactions between species and the aforementioned factors). We also measured a suite of soil abiotic and biotic properties to help explain the impacts of maternal effects, drought legacy and diversity legacy effects on plant performance.

## 2 MATERIALS AND METHODS

### 2.1 Study site and field experimental set‐up

Soils and seeds for this experiment were collected from one site in Selside Shaw Meadow (54°10′47.9″N, 2°20′11.1″W), Ingleborough National Nature Reserve, England. The soils are brown earth (De Deyn, Shiel, et al., 2011; 60% clay, 39% sand, <1% silt, pH ~5.7, 4.9% C, 0.46% N), and the site has received a light application of farmyard manure and/or 20:10:10 NPK fertilizer annually for several decades. Sheep or cattle graze the site from autumn to spring, and a hay crop is taken each summer. The elevation is 303 m a.s.l., annual average daily min and max temperatures between 1981 and 2010 were 4.3 and 10.5°C, respectively, and average annual precipitation was 1,550 mm (measured at Malham Tarn climate station 18 km from the site; www.metoffice.gov.uk). The species composition is typical of species‐rich meadow communities of northern pastures (UK National Vegetation Classification MG3b; Rodwell, 1998).

Plots used for seed and soil collection were part of an experimental set‐up in summer 2012 and described in Leff et al. (2018) and De Long, Jackson, et al. (2019). Briefly, plots were assigned to plant functional group addition treatments (control, grass, forb, legumes) in a fully factorial randomized block design. Seeds and seedlings of the target plant functional groups were added to the assigned plots each year from 2013 to 2014 to increase plant species diversity (Supporting Information Tables S1–S3). The two focal species of this study, *H. lanatus* and *A. pratensis*, were not included in the functional group addition treatments. Plots were not weeded to maintain specific species assemblages. We selected the control (i.e. no plant species additions; hereafter low diversity) and the most species‐rich treatment (i.e. addition of grasses, forbs and legumes; hereafter high diversity). Four years later (2016), at the time of sampling, the experimental addition of grasses, forbs and legumes had increased both plot species richness (number of species) (*p* < 0.001; *t*‐value = 12.0) and diversity (Shannon–Wiener index; *p* < 0.001; *t*‐value = 9.4) from 20.8 ± 1.4 and 2.5 ± 0.1, respectively, in the low diversity plots, to 34.6 ± 2.0 and 2.9 ± 0.08, respectively, in the high diversity plots.

**Figure 1:**
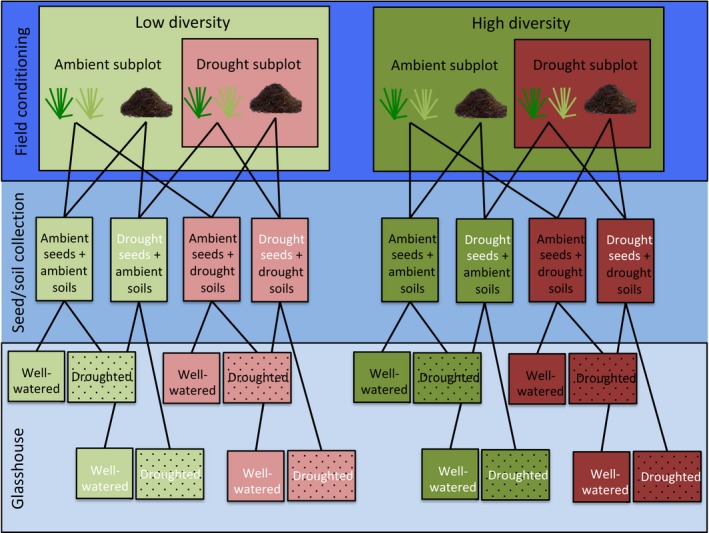
Schematic depiction of the experimental design. In the field, seeds from one subordinate [*Alopecurus pratensis*] and one dominant [*Holcus lunatus*] grass species and soils were collected from both ambient and drought subplots within both low and high plant diversity plots (*n* = 5 for both high and low diversity plots, yielding a total of 20 subplots from which soils and seeds were collected). Next, seeds from both species from both ambient and drought origin were planted into pots containing ambient soils and drought legacy soils taken from the same field plot (one individual per pot). During the glasshouse experiment, half of the pots from each treatment combination were well‐watered and half were droughted for 4 weeks

On 15 May 2016, drought shelters were set up in subplots within each of the plots, where they remained until 27 July 2016. Shelters covered an area of 2 × 2 m and had a height of 1.0 m to allow for air movement and to reduce greenhouse effects (Beier et al., 2012). Shelters were angled with the slope of the field to ensure proper run off of rainwater. The plastic used was NUDEC^®^ polymethylmethacrylate (2 mm thickness). This plastic has a higher light transmission than the corrugated PVC roofing sheets typically used to construct drought shelters (92% light transmission vs. 85%) (Vogel, Fester, et al., 2013). Therefore, our shelters helped control for artefact effects of reduced light transmission (Beier et al., 2012; Vogel, Fester, et al., 2013). Low diversity ambient and drought subplots had average soil water contents of 59.1 ± 1.4% and 24.3 ± 0.7%, respectively, and high diversity ambient and drought subplots had average soil water contents of 61.8 ± 1.5% and 24.4 ± 0.6%, respectively. All subsequent vegetation and soil measurements were taken at minimum 30 cm from the edges of the drought subplot to avoid edge effects. For this experiment, we collected seeds and soils from subplots that had been exposed to both ambient and drought treatments from both the low and high diversity plots (*n* = 5 for each treatment; total subplots sampled *n* = 20; Figure 1).

### 2.2 Glasshouse experimental set‐up

Seeds of the two focal grass species, *H. lanatus* (dominant: 15.6 ± 1.6% cover) and *A. pratensis* (subordinate: 2.6 ± 0.3% cover), were collected from subplots that had been exposed to ambient and drought treatments in both low and high diversity plots on 27 July and 17 August 2016 (Figure 1). Seeds of both species were collected at two different times due to timing of seed ripening (i.e. certain plants of both species ripened earlier than others). After collection, seeds were transported back to the laboratory and dried at room temperature (25°C) until 27 September 2016 when they were placed in the refrigerator (4°C) for two weeks to cold stratify the seeds. Subsamples of seeds from each species and seed origin combination were weighed and analysed for %C and %N to determine whether seed weight or nutrient content potentially affected plant performance and maternal effects (Dyer et al., 2010; Germain, Caruso, & Maherali, 2013). On 11 October 2016, seeds were surface‐sterilized using a 1% sodium hypochlorite solution for 1 min, rinsed, sown in autoclaved sand and placed in a growth chamber (18°C, 16‐hr light, 8‐hr dark).

In order to test for drought and plant diversity legacy effects, soils were collected from the field on 4 November 2016 from each ambient and drought treatment subplot within each of the low and high diversity plots (Figure 1). A clean, sterilized spade was used to turn over the turf and soil was removed from the surface 10 cm of topsoil, which contained roots, thereby ensuring the soil collected was sufficiently conditioned by the rhizosphere of the plant community. A total of 6 L of soil was collected from each subplot. Soils were transported back to the laboratory and kept at 4°C until the experiment was set up.

From 7 to 8 November 2016, subsamples of soil from within each field treatment combination from each individual plot (i.e. both ambient and drought legacy treatment subplots within the low and high diversity plots) were carefully homogenized, large stones and roots were removed, and soils were placed into plastic pots (cylindrical with a total volume of 600 ml; H: 9.6 cm, diameter: 11.5 cm, tapering to 7.2 cm). Each pot contained a piece of filter paper and a drainage layer at the bottom of 150 ml of sterilized sand and the remaining volume filled with the collected field soil.

Replicate pots were filled with soil from each diversity legacy treatment × drought legacy treatment so that one pot could be exposed to control moisture conditions and the other could be exposed to drought conditions (see end of paragraph) (Figure 1). On 9 November 2016, one seedling of each grass species (*A. pratensis* and *H. lanatus*) from each diversity soil legacy and drought soil legacy treatment combination was transplanted into each diversity legacy × drought legacy treatment combination. Specifically, one seedling of each species was planted into its respective soil (e.g. seedlings that came from drought subplots were planted back into soils from the same droughted subplot) and one seedling was planted into a different drought legacy soil from within the same plot (e.g. seedlings that came from a drought subplot were planted into soils from the corresponding ambient subplots). In total, this created five treatments: maternal origin (ambient maternal, drought maternal), drought legacy (ambient legacy, drought legacy), diversity legacy (low diversity, high diversity), glasshouse watering (well‐watered, droughted) and species (*A. pratensis* and *H. lanatus*). With five replicates, this design resulted in 160 pots (Figure 1).

After transplanting, seedlings were watered as necessary with tap water for ten weeks before the onset of the experimental drought (*A. pratensis* and *H. lanatus* were on average 29.3 ± 1.0 and 26.1 ± 0.5 cm tall, respectively, at the time of drought onset). Pots were weeded as necessary. On 23 January 2017, the glasshouse drought began. Well‐watered pots were kept at 70% water holding capacity (WHC) and droughted pots were kept at 30% WHC for 4 weeks, which is comparable to conditions experienced in the field (see above). Pots were weighed every other day, and the appropriate amount of water was added to keep them at the designated WHC. Afterwards, pots were all watered to 70% WHC for 2 weeks to ensure that the plants and soils were recovered sufficiently and able to process the added ^15^N solution.

Three days before the experiment was harvested, 25 ml of ^15^NH_4_^15^NO_3_ solution (98 atom % excess, 3.93 mg ^15^N per pot; Sigma‐Aldrich) was injected in the top 5 cm of each pot at 5, evenly spaced locations (5 ml in each location), following de Vries et al. (2012). This enabled us to test how maternal environment and soil legacies affected short‐term plant acquisition and distribution (i.e. roots and shoots) of soil mineral N.

### 2.3 Glasshouse experimental harvest

After 117 days of growth, the experiment was destructively harvested on 6 March 2017. Plants were carefully removed from each pot, and the layer of sand was separated from the rest of the soil and discarded. All soil was sieved (4 mm) for further analyses (see below). The roots of each plant were washed using tap water to remove soil and divided into shoot and root biomass. A subsample of each root system was weighed and stored in 50% ethanol at 4°C for mycorrhizal colonization assessment (see below). Shoots and the remaining roots were dried at 40°C for 72 hr and weighed. Subsamples of dried material were ground (Retsch Ball Mill MM 400) and analysed for C and N concentrations (%) and δ^15^N isotope ratios by James Hutton Ltd., using a Flash EA 1112 Series Elemental Analyser connected via a Conflo III to a DeltaPlus XP isotope ratio mass spectrometer (Thermo Fisher Scientific). The δ^15^NAir‐N_2_ values were normalized using International Atomic Energy Agency reference materials USGS40 and USGS41a (both L‐glutamic acid). The USGS40 was used as a reference material for N concentrations, measured using the area output of the mass spectrometer.

Roots were cleared with 2.5% KOH, stained with acidic glycerol solution containing 0.05% trypan blue (Sigma Co.; Koske & Gemma, 1989), and colonization of hyphae, arbuscules and vesicles was measured using the gridline intersection method (i.e. structures intersecting microscope lens crosshairs; McGonigle, Miller, Evans, Fairchild, & Swan, 1990).

### 2.4 Soil abiotic and biotic properties

We measured soil abiotic and biotic properties to help explain the impacts of maternal, drought legacy and diversity legacy effects on plant performance. A total of 5 g (wet weight) soil was extracted with 25 ml of 1 M KCl. Extracts were analysed immediately for total inorganic nitrogen (TIN) on Seal AA3 Segmented Flow Multi‐chemistry analyser. Microbial biomass C and N were measured using the chloroform fumigation (Brookes, Landman, Pruden, & Jenkinson, 1985). Soil extracellular enzyme (i.e. amino acid deaminase [DEA], glucosidase [GLC], N‐acetylglucosaminidase [NAG], peroxidase [PER], phenoloxidase [POX], phosphatase [PHO], urease [URE] and xylosidase [XYL]) activities were measured using photometric assays. See Supporting Information Appendix S1 for details.

### 2.5 Statistical analyses

All plant and soil data from the glasshouse experiment were analysed using linear mixed effects models. Diversity legacy (low, high), drought legacy (ambient legacy, drought legacy), maternal origin (ambient maternal, drought maternal), species (*A. pratensis* and *H. lanatus*) and glasshouse watering (well‐water, droughted; hereafter watering) were included as fixed effects, with all three‐way interactions specified. We used restricted maximum likelihood and type III sum of squares in all models. Simulations show that REML method with Kenward–Roger or Satterthwaite approximations for degrees of freedom produces acceptable type I error rates and performs well even with small sample sizes (Luke, 2017). We used Kenward–Roger approximation for degrees of freedom as our method of inference. Higher order interactions (i.e. 4‐way and 5‐way) were not included in the models because they were not necessary to address the hypotheses specified in the Introduction, which primarily focused on two‐ and three‐way interactions, and higher order interactions are likely to be subject to high type I and II errors (Smith, Levine, Lachlan, & Fediuk, 2002). Field block (i.e. blocks of the experimental design in the field), plot (i.e. each field plot that was divided into two subplots: control and drought) and glasshouse block (i.e. the randomized block design into which all the pots were placed in the glasshouse) were included as random effects, with plot nested within field block, crossed with glasshouse block. Seed weight and C and N data were analysed using mixed effects models, with diversity, maternal origin and species as fixed factors and random factors as above. Whenever significant interactions were detected, post hoc tests were performed using the *lsmeans* package in R (Lenth, 2016) with Tukey HSD adjustment, which accounts for multiple comparisons. All data were transformed as necessary to meet the model assumptions (see ANOVA tables for details; Supporting Information Tables S4A–S7B). Analyses were performed using R software (R Core Team, 2015) with the package *lme4* (Bates, Mächler, Bolker, & Walker, 2015).

## 3 RESULTS

### 3.1 Maternal and drought legacy effects

**Figure 2:**
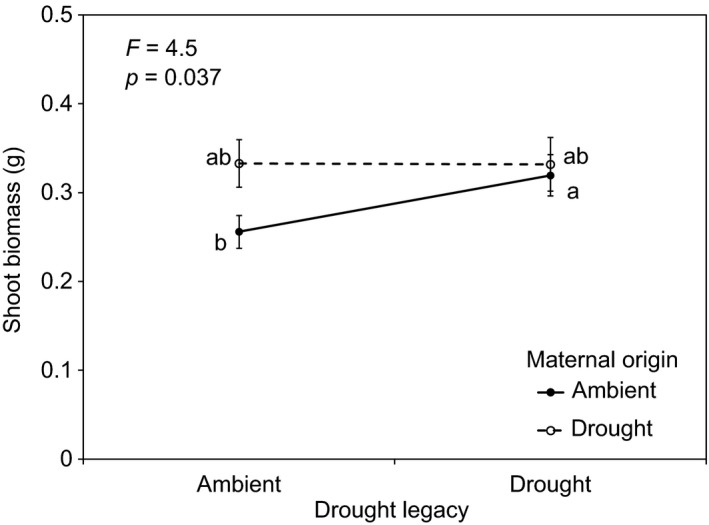
Shoot biomass averaged across all plants (*Alopecurus pratensis* and *Holcus lanatus*) from seeds of ambient and drought maternal origin grown in ambient and drought legacy soils. Points accompanied by the same lower case letter do not differ at *p* < 0.05 (Tukey's HSD). Data are means plus one standard error (*n* = 138). ANOVA results are presented in Supporting Information Tables S4a,b

**Figure 3:**
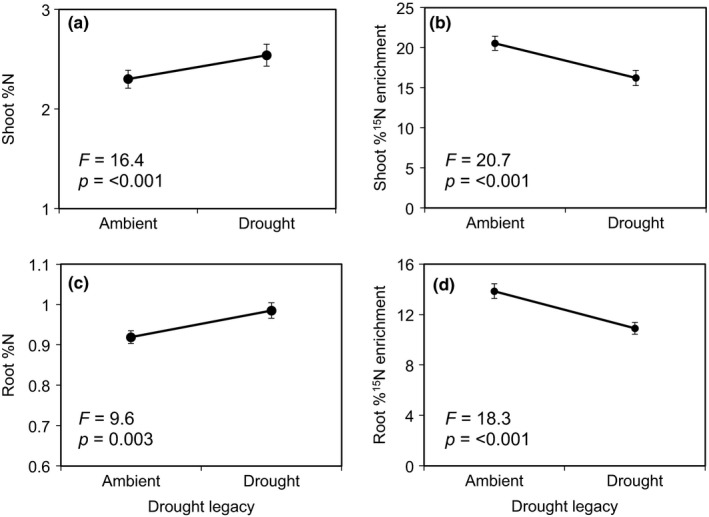
Shoot nitrogen (%N) content of plants (panel a); shoot atom %^15^N excess (panel b); root %N content (panel c); and root atom %^15^N excess (panel d) of plants (averaged across *Alopecurus pratensis* and *Holcus lanatus*) grown in soils from ambient and drought legacies. Within each panel that contains more than two bars, bars topped with the same lower case letter do not differ at *p* < 0.05 (Tukey's HSD). Data are means ± 1 *SE* (shoot %N and shoot atom %^15^N excess, root %N: *n* = 138, root atom ^15^N excess: *n* = 136). ANOVA results are presented in Supporting Information Tables S5a,b

**Figure 4:**
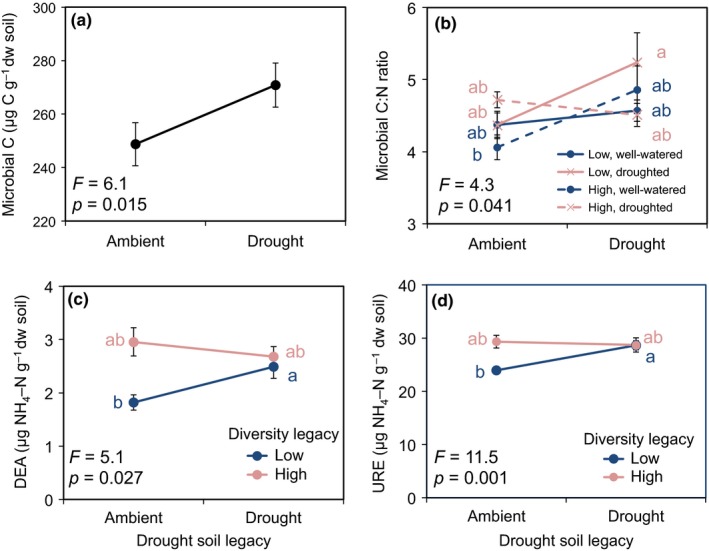
Microbial C (carbon) (panel a) of soils from ambient and drought legacy; microbial carbon‐to‐nitrogen (C:N) ratios (panel b) of soils from ambient and drought legacy, low and high diversity legacy soils that were well‐watered and droughted; amino acid deaminase (DEA) (panel c) and urease (URE) (panel d) measurements of soils from low and high diversity legacy, and ambient and drought legacy. Data are averaged across the two study species (*Alopecurus pratensis*, *Holcus lanatus*). Within each panel, points accompanied by the same lower case letter do not differ at *p* < 0.05 (Tukey's HSD). Data are means ± 1 *SE* (*n* = 138). ANOVA results are presented in Supporting Information Tables S6 and 7Sa,b

Shoot biomass was 20% lower in plants from ambient maternal origin when grown in soils with ambient legacy compared to drought legacy; plants from drought maternal origin showed no significant difference in shoot biomass between ambient and drought legacy soils (maternal effect × drought legacy interaction, Figure 2). Root and shoot %N were 7% and 10% higher, respectively, in plants grown in drought vs. ambient legacy soils (Figure 3a,c). Both root and shoot atom ^15^N excess were 21% lower in plants grown in drought vs. ambient legacy soils (Figure 3b,d). Microbial C was significantly higher in drought legacy vs. ambient legacy soils (Figure 4a). Seed %C and %N were not affected by maternal origin or drought legacy (Supporting Information Appendix S2).

### 3.2 Diversity legacy interactions with maternal effects and drought legacy

Shoot biomass and hyphae and arbuscule colonization were affected by maternal effect and diversity legacy interactions, but post hoc tests revealed no significant differences between treatments (Supporting Information Tables S4a,b; means not shown). The enzymes DEA and URE were both higher in drought than ambient low diversity legacy soils, but this effect disappeared in high diversity soils (diversity × drought legacy interaction; Figure 4c,d).

### 3.3 Species interactions with maternal effects, drought legacy and diversity legacy

**Figure 5:**
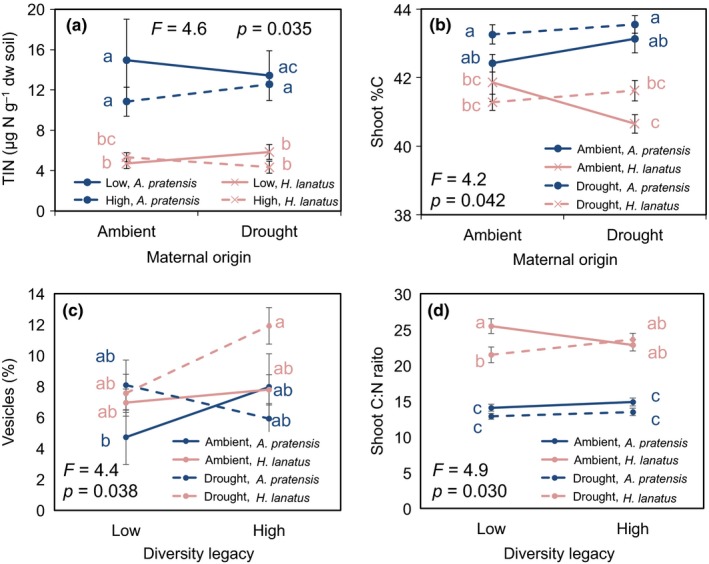
Total inorganic nitrogen (TIN) measurements (panel a) from *Alopecurus pratensis* and *Holcus lanatu*s grown in low and high diversity legacy, ambient and drought legacy soils; shoot %C (panel b) of *Alopecurus pratensis* and *Holcus lanatus* from ambient and drought maternal origin grown in soils from ambient and drought legacy; mycorrhizal vesicle % colonization (panel c) of *Alopecurus pratensis* and *Holcus lanatus* grown in soils from ambient and drought legacy, low and high diversity legacy soils and shoot carbon‐to‐nitrogen (C:N) ratios (panel d) of *Alopecurus pratensis* and *Holcus lanatus* grown in soils from ambient and drought legacy, low and high diversity legacy soils. Within each panel, points accompanied by the same lower case letter do not differ at *p* < 0.05 (Tukey's HSD). Data are means ± 1 *SE* (*n* = 138 except total inorganic N: *n* = 137). ANOVA results are presented in Supporting Information Tables S4–S6a,b

Vesicle colonization was higher in *H. lanatus* plants grown on high diversity drought legacy soils compared to *A. pratensis* plants grown on low diversity, ambient legacy soils (diversity × drought legacy × species interaction, Figure 5c). Shoot %C was higher in *A. pratensis* than *H. lanatus* when seeds came from ambient and drought maternal origin and were grown in drought legacy soils, but this effect disappeared when seeds came from ambient maternal origin and were grown in ambient legacy soils (maternal effect × drought legacy × species interaction, Figure 5b). Shoot C:N ratios were higher in *H. lanatus* grown in low diversity ambient vs. drought legacy soils, but this effect disappeared in high diversity soils and no differences were detected in *A. pratensis* (drought legacy × diversity legacy × species interaction; Figure 5d).

Microbial C:N ratios were higher in low diversity drought legacy soils that were droughted compared to high diversity ambient legacy soils that were also droughted (diversity × drought legacy × watering interaction; Figure 4b). Regardless of diversity legacy, TIN was higher in soil planted with *A. pratensis* than *H. lanatus*. However, this effect disappeared when *A. pratensis* seeds came from drought maternal origin and were grown on low diversity legacy soils and when *H. lanatus* seeds came from ambient maternal origin and were grown on high diversity legacy soils (maternal effect × diversity legacy × species interaction; Figure 5a). Microbial C:N ratios were affected by drought and diversity legacy and species interactions, but post hoc tests revealed no significant differences between treatments (drought legacy × diversity legacy × species interaction; means not shown). Finally, there was a significant drought legacy × diversity legacy × species interaction on GLC and a significant maternal × drought legacy × species interaction on the enzyme PHO, but, again, post hoc tests revealed no differences between treatments (means not shown). More details on significant effects and means not discussed in the text or displayed in the Figures are in Supporting Information Appendix S2 and Table S8, respectively.

## 4 DISCUSSION

Our goal was to evaluate interactions between drought maternal effects and drought and diversity soil legacies on grassland plant performance. Although we detected significant drought maternal effects on shoot biomass, drought soil legacy cancelled out these effects due to changes in soil properties. These results suggest that drought soil legacy effects, which are likely to increase in extent and relative strength with climate change, have stronger impacts on plant performance than drought maternal effects. Further, diversity legacy altered some of the maternal and drought legacy effects on plant and soil microbial properties. Such effects could have longer‐term consequences for plant community dynamics and ecosystem function in grasslands that experience drought.

### 4.1 Maternal and drought legacy effects

Our first prediction was not supported because we did not find drought maternal effects that helped to improve plant performance under glasshouse drought conditions. This suggests that droughted mother plants may not gain a fitness advantage from pre‐adapting their offspring to subsequent drought, because drought events are highly unpredictable in their nature (Metz, von Oppen, & Tielborger, 2015). It is predictable, however, that drought legacy can lead to nutrient pulses once soils are rewetted (Birch, 1958; Bloor & Bardgett, 2012; Leitner et al., 2017). Therefore, faster growth and development following drought might enhance a plant's ability to utilize post‐drought nutrient flushes, with maternal drought effects allowing plants to out‐compete their neighbours.

In line with this prediction, we found that drought soil legacy affected shoot biomass, shoot and root N content, and atom ^15^N excess in shoots and roots, lending support to our second prediction that plants would respond to drought legacy effects. Plants were larger and had higher shoot and root N concentrations when grown in drought legacy soils. Many studies have shown that post‐drought soils have higher nutrient concentrations due to a pulse of mineralization following rewetting, which is primarily caused by the reactivation of microbial activity that leads to the decomposition of dead microbial and plant biomass (Birch, 1958; Bloor & Bardgett, 2012; Leitner et al., 2017). In line with this mechanism, drought legacy resulted in an increase in microbial biomass and soil inorganic N, as well as increased urease enzyme activity, which together likely contributed to higher plant biomass in drought legacy soils. In addition, plants grown in drought legacy soils took up less ^15^N than those grown in ambient legacy soils, but plants grown in drought legacy soils had higher tissue N concentrations. This was likely because plants were able to capitalize on the post‐drought nutrient flush early on (Birch, 1958; Bloor & Bardgett, 2012; Leitner et al., 2017), but once the microbial community recovered, it likely immobilized any additional N (i.e. the added ^15^N). Considered collectively, if drought events become more common with advancing global climate change, drought legacies that result in flushes of N might help compensate for the deleterious effects of water shortage, thereby helping to maintain plant productivity under recurrent droughts (Arredondo et al., 2016).

Our second prediction was also supported because drought legacy interacted with maternal effects. However, instead of the enhanced offspring performance in soil conditions experienced by their mothers, plants of drought maternal origin performed better than plants of ambient maternal origin when grown on ambient soils. However, plants of the two contrasting maternal origins performed equally when grown on drought legacy soils. Drought legacy soils contained more plant‐available N, which may have allowed the ambient maternal origin plants to match the growth of the drought maternal origin plants. This experiment was conducted in the absence of competition, which probably affected maternal and soil drought legacy effects and their interactions. However, our findings suggest that if seeds germinate in a more N‐poor environment than their parents grew, they could out‐compete their neighbours by taking up more N (Kaisermann et al., 2017). On the other hand, if drought maternal origin seeds germinate in N‐rich environments, any advantage in nutrient uptake conveyed by the drought maternal effect is negated. This is because N was already in surplus due to the post‐drought nutrient flush. Taken collectively, our findings support other studies that demonstrate maternal effects are important moderators of plant performance (Galloway & Etterson, 2007; Mousseau et al., 2009; Rottstock et al., 2017). However, our results question the importance of maternal effects for shaping plant community interactions, particularly under climate change scenarios (Münzbergová & Hadincová, 2017; Walter et al., 2016). Our data suggest that drought soil legacy effects might be stronger than maternal effects in driving plant performance in temperate grasslands as drought events become more frequent and intense with climate change.

### 4.2 Diversity legacy effect interactions with maternal effects and drought legacy

Our third prediction was partially supported, as plant diversity legacy interacted with maternal effects or drought legacy. There were significant interactions between diversity and drought legacies: the enzymes DEA (which breaks down amino acids, thereby releasing ammonia) and URE (which degrades urea and is considered a proxy for N mineralization) were consistently more active and stable in high diversity soils and less active in low diversity soils. Their activity was also lowest in ambient soils, but significantly increased in drought legacy soils. These responses may be because low diversity drought legacy soils had increased microbial activity due to post‐drought N flushes (Birch, 1958; Bloor & Bardgett, 2012; Leitner et al., 2017), while enzymes were already more active in high diversity legacy soils due to competing nutrient demands of a more diverse soil community (Steinauer et al., 2015).

### 4.3 Species interactions with maternal effects, drought legacy and diversity legacy

Our fourth prediction was partially supported as the two plant species responded differently to maternal effects and drought and diversity legacy effects. In soils with low plant diversity legacy, shoot C:N ratios were lower in *H. lanatus* plants grown in soil from drought compared with ambient legacy subplots, but these effects were not detected for *A. pratensis*. This likely reflects the greater capacity of *H. lanatus* to respond to higher N availability in drought legacy soils (Weigelt, Bol, & Bardgett, 2005). Further, *H. lanatus* roots were colonized more heavily by mycorrhizal fungi, particularly when grown in high diversity drought legacy soils, and together, these findings indicate a propensity of *H. lanatus* to invest in resource acquisitive traits under contrasting conditions. This inherent adaptability of *H. lanatus* to variable soil conditions could partially explain why this species is able to maintain dominance in many grassland habitats. On the other hand, shoot C was higher for *A. pratensis* than *H. lanatus*, except when *A. pratensis* came from ambient maternal origin and was grown on ambient legacy soils. Higher shoot C could be the result of higher concentrations of structural defence compounds, which may have been upregulated in *A. pratensis* in response to environmental stressors (Caretto, Linsalata, Colella, Mita, & Lattanzio, 2015). Therefore, when *A. pratensis* came from an ambient maternal environment and were grown in ambient legacy soils (i.e. less stressful conditions), these secondary defence compounds were downregulated. This was probably due to maternal effects (Mousseau et al., 2009) and the deleterious effects of drought legacy mediated via the soil microbial community (Kaisermann et al., 2017). This strategy may be what allows *A. pratensis* to persist as a subordinate species in grassland subjected to stressors.

## 5 CONCLUSIONS

Drought events can affect soil properties including nutrient availability and microbial community composition (Bloor & Bardgett, 2012; Kaisermann et al., 2017; Leitner et al., 2017), which can alter plant productivity (Arredondo et al., 2016). Here, we show that a single drought event in the field can also create significant maternal and soil legacy effects, which translated to improved growth of two common grass species. Drought maternal or soil legacy effects could have positive implications for the capacity of plant communities to resist and recover from future drought events (Backhaus et al., 2014). However, we demonstrate that drought legacy effects on soil are more important for plant performance than are drought maternal effects. This suggests that, at least in the short term, drought soil legacy effects have the greatest potential to influence grassland community dynamics and ecosystem function. Despite this, caution must be exercised in drawing broad conclusions, as the effects of drought can vary depending on timing, duration and intensity (Hoover & Rogers, 2016), and variation in the tolerance of grass species to drought (Craine et al., 2013), which might ultimately lead to contrasting consequences for plant fitness. Further, repeated or more intense drought events (Fuchslueger et al., 2016; Kopittke, Tietema, van Loon, & Asscheman, 2014) could generate contrasting responses between species or even reorder the relative importance of maternal vs. soil legacy effects. Nevertheless, our findings pull focus on the need to consider interactions between the maternal and soil legacy effects of climatic extremes, such as drought, on plant performance and ecosystem function in temperate grasslands. Lastly, we found that plant community diversity modified plant and soil microbial responses to drought legacies, highlighting the potential for plant diversity to mitigate some of the negative impacts of extreme weather events. Considering how drought soil legacy effects can override maternal effects will allow us to better predict how grassland plant communities might respond as climate extremes, such as drought, become more common.

## AUTHORS' CONTRIBUTIONS

J.R.D., M.S. and R.D.B. conceived and designed the study, using funds gained by R.D.B.; J.R.D., W.J.P., E.L.F., I.C. and K.K. collected the data; J.R.D., E.L.F. and B.G.J. analysed the data; and J.R.D. led the writing of the manuscript. All authors contributed to the drafts and gave final approval for publication.

## DATA ACCESSIBILITY

Data are deposited in the Dryad Digital Repository https://doi.org/10.5061/dryad.7j43s83 (De Long, Semchenko, et al., 2019).

## References

[fec13341-bib-0001] Arredondo, T. , Garcia‐Moya, E. , Huber‐Sannwald, E. , Loescher, H. W. , Delgado‐Balbuena, J. , & Luna‐Luna, M. (2016). Drought manipulation and its direct and legacy effects on productivity of a monodominant and mixed‐species semi‐arid grassland. Agricultural and Forest Meteorology, 223, 132–140. 10.1016/j.agrformet.2016.03.011

[fec13341-bib-0002] Backhaus, S. , Kreyling, J. , Grant, K. , Beierkuhnlein, C. , Walter, J. , & Jentsch, A. (2014). Recurrent mild drought events increase resistance toward extreme drought stress. Ecosystems, 17, 1068–1081. 10.1007/s10021-014-9781-5

[fec13341-bib-0003] Bates, D. , Mächler, M. , Bolker, B. , & Walker, S. (2015). Fitting linear mixed‐effects models using lme4. Journal of Statistical Software, 67, 1–48. 10.18637/jss.v067.i01

[fec13341-bib-0004] Beier, C. , Beierkuhnlein, C. , Wohlgemuth, T. , Penuelas, J. , Emmett, B. , Körner, C. , … Hansen, K. (2012). Precipitation manipulation experiments – Challenges and recommendations for the future. Ecology Letters, 15, 899–911. 10.1111/j.1461-0248.2012.01793.x 22553898

[fec13341-bib-0005] Birch, H. F. (1958). The effect of soil drying on humus decomposition and nitrogen availability. Plant and Soil, 10, 9–31. 10.1007/bf01343734

[fec13341-bib-0006] Bloor, J. M. G. , & Bardgett, R. D. (2012). Stability of above‐ground and below‐ground processes to extreme drought in model grassland ecosystems: Interactions with plant species diversity and soil nitrogen availability. Perspectives in Plant Ecology Evolution and Systematics, 14, 193–204. 10.1016/j.ppees.2011.12.001

[fec13341-bib-0007] Brookes, P. C. , Landman, A. , Pruden, G. , & Jenkinson, D. S. (1985). Chloroform fumigation and the release of soil‐nitrogen – A rapid direct extraction method to measure microbial biomass nitrogen in soil. Soil Biology & Biochemistry, 17, 837–842. 10.1016/0038-0717(85)90144-0

[fec13341-bib-0008] Burgess, S. C. , & Marshall, D. J. (2014). Adaptive parental effects: The importance of estimating environmental predictability and offspring fitness appropriately. Oikos, 123, 769–776. 10.1111/oik.01235

[fec13341-bib-0009] Caretto, S. , Linsalata, V. , Colella, G. , Mita, G. , & Lattanzio, V. (2015). Carbon fluxes between primary metabolism and phenolic pathway in plant tissues under stress. International Journal of Molecular Sciences, 16, 26378–26394. 10.3390/ijms161125967 26556338 PMC4661826

[fec13341-bib-0010] Craine, J. M. , Ocheltree, T. W. , Nippert, J. B. , Towne, E. G. , Skibbe, A. M. , Kembel, S. W. , & Fargione, J. E. (2013). Global diversity of drought tolerance and grassland climate‐change resilience. Nature Climate Change, 3, 63–67. 10.1038/nclimate1634

[fec13341-bib-0011] Craven, D. , Isbell, F. , Manning, P. , Connolly, J. , Bruelheide, H. , Ebeling, A. , … Eisenhauer, N. (2016). Plant diversity effects on grassland productivity are robust to both nutrient enrichment and drought. Philosophical Transactions of the Royal Society B‐Biological Sciences, 371, 8 10.1098/rstb.2015.0277 PMC4843698 27114579

[fec13341-bib-0012] De Deyn, G. B. , Quirk, H. , & Bardgett, R. D. (2011). Plant species richness, identity and productivity differentially influence key groups of microbes in grassland soils of contrasting fertility. Biology Letters, 7, 75–78. 10.1098/rsbl.2010.0575 20685699 PMC3030891

[fec13341-bib-0013] De Deyn, G. B. , Shiel, R. S. , Ostle, N. J. , McNamara, N. P. , Oakley, S. , Young, I. , … Bardgett, R. D. (2011). Additional carbon sequestration benefits of grassland diversity restoration. Journal of Applied Ecology, 48, 600–608. 10.1111/j.1365-2664.2010.01925.x

[fec13341-bib-0014] De Long, J. R. , Jackson, B. G. , Wilkinson, A. , Pritchard, W. J. , Oakley, S. , Mason, K. E. , … Bardgett, R. D. (2019). Relationships between plant traits, soil properties and carbon fluxes differ between monocultures and mixed communities in temperate grassland. Journal of Ecology, 107, 1400–1410. 10.1111/1365-2745.13160 PMC6617750 31341333

[fec13341-bib-0015] De Long, J. R. , Kardol, P. , Sundqvist, M. K. , Veen, G. F. , & Wardle, D. A. (2015). Plant growth response to direct and indirect temperature effects varies by vegetation type and elevation in a subarctic tundra. Oikos, 124, 772–783. 10.1111/oik.01764

[fec13341-bib-0016] De Long, J. R. , Semchenko, M. , Pritchard, W. J. , Cordero, I. , Fry, E. L. , Jackson, B. G. , … Bardgett, R. D. (2019). Data from: Drought soil legacy overrides maternal effects on plant growth. Dryad Digital Repository,. 10.5061/dryad.7j43s83 PMC6767434 31588158

[fec13341-bib-0017] de Vries, F. T. , Bloem, J. , Quirk, H. , Stevens, C. J. , Bol, R. , & Bardgett, R. D. (2012). Extensive management promotes plant and microbial nitrogen retention in temperate grassland. PLoS ONE, 7, 1–12. 10.1371/journal.pone.0051201 PMC3515579 23227252

[fec13341-bib-0018] Dyer, A. R. , Brown, C. S. , Espeland, E. K. , McKay, J. K. , Meimberg, H. , & Rice, K. J. (2010). The role of adaptive trans‐generational plasticity in biological invasions of plants. Evolutionary Applications, 3, 179–192. 10.1111/j.1752-4571.2010.00118.x 25567918 PMC3352481

[fec13341-bib-0019] Evans, S. E. , & Wallenstein, M. D. (2012). Soil microbial community response to drying and rewetting stress: Does historical precipitation regime matter? Biogeochemistry, 109, 101–116. 10.1007/s10533-011-9638-3

[fec13341-bib-0020] Fuchslueger, L. , Bahn, M. , Hasibeder, R. , Kienzl, S. , Fritz, K. , Schmitt, M. , … Richter, A. (2016). Drought history affects grassland plant and microbial carbon turnover during and after a subsequent drought event. Journal of Ecology, 104, 1453–1465. 10.1111/1365-2745.12593 27609992 PMC4996329

[fec13341-bib-0021] Galloway, L. F. , & Etterson, J. R. (2007). Transgenerational plasticity is adaptive in the wild. Science, 318, 1134–1136. 10.1126/science.1148766 18006745

[fec13341-bib-0022] Germain, R. M. , Caruso, C. M. , & Maherali, H. (2013). Mechanisms and consequences of water stress‐induced parental effects in an invasive annual grass. International Journal of Plant Sciences, 174, 886–895. 10.1086/670691

[fec13341-bib-0023] Herman, J. J. , & Sultan, S. E. (2011). Adaptive transgenerational plasticity in plants: Case studies, mechanisms, and implications for natural populations. Frontiers in Plant Science, 2, 1–10. 10.3389/fpls.2011.00102 22639624 PMC3355592

[fec13341-bib-0024] Herman, J. J. , Sultan, S. E. , Horgan‐Kobelski, T. , & Riggs, C. (2012). Adaptive transgenerational plasticity in an annual plant: Grandparental and parental drought stress enhance performance of seedlings in dry soil. Integrative and Comparative Biology, 52, 77–88. 10.1093/icb/ics041 22523124

[fec13341-bib-0025] Hoover, D. L. , & Rogers, B. M. (2016). Not all droughts are created equal: The impacts of interannual drought pattern and magnitude on grassland carbon cycling. Global Change Biology, 22, 1809–1820. 10.1111/gcb.13161 26568424

[fec13341-bib-0026] Hovenden, M. J. , Wills, K. E. , Chaplin, R. E. , Schoor, J. K. V. , Williams, A. L. , Osanai, Y. , & Newton, P. C. D. (2008). Warming and elevated CO2 affect the relationship between seed mass, germinability and seedling growth in *Austrodanthonia caespitosa*, a dominant Australian grass. Global Change Biology, 14, 1633–1641. 10.1111/j.1365-2486.2008.01597.x

[fec13341-bib-0027] Kaisermann, A. , de Vries, F. T. , Griffiths, R. I. , & Bardgett, R. D. (2017). Legacy effects of drought on plant–soil feedbacks and plant–plant interactions. New Phytologist, 215, 1413–1424. 10.1111/nph.14661 28621813

[fec13341-bib-0028] Kopittke, G. R. , Tietema, A. , van Loon, E. E. , & Asscheman, D. (2014). Fourteen annually repeated droughts suppressed autotrophic soil respiration and resulted in an ecosystem change. Ecosystems, 17, 242–257. 10.1007/s10021-013-9720-x

[fec13341-bib-0029] Koske, R. E. , & Gemma, J. N. (1989). A modified procedure for staining roots to detect VA‐mycorrhizas. Mycological Research, 92, 486–505. 10.1016/S0953-7562(89)80195-9

[fec13341-bib-0030] Leff, J. W. , Bardgett, R. D. , Wilkinson, A. , Jackson, B. G. , Pritchard, W. J. , De Long, J. R. , … Fierer, N. (2018). Predicting the structure of soil communities from plant community taxonomy, phylogeny, and traits. The ISME Journal, 12(7), 1794–1805. 10.1038/s41396-018-0089-x 29523892 PMC6004312

[fec13341-bib-0031] Leitner, S. , Homyak, P. M. , Blankinship, J. C. , Eberwein, J. , Jenerette, G. D. , Zechmeister‐Boltenstern, S. , & Schimel, J. P. (2017). Linking NO and N2O emission pulses with the mobilization of mineral and organic N upon rewetting dry soils. Soil Biology & Biochemistry, 115, 461–466. 10.1016/j.soilbio.2017.09.005

[fec13341-bib-0032] Lenth, R. V. (2016). Least‐squares means: The R package lsmeans. Journal of Statistical Software, 69, 33 10.18637/jss.v069.i01

[fec13341-bib-0033] Luke, S. G. (2017). Evaluating significance in linear mixed‐effects models in R. Behavior Research Methods, 49, 1494–1502. 10.3758/s13428-016-0809-y 27620283

[fec13341-bib-0034] Mariotte, P. , Robroek, B. J. M. , Jassey, V. E. J. , & Buttler, A. (2015). Subordinate plants mitigate drought effects on soil ecosystem processes by stimulating fungi. Functional Ecology, 29, 1578–1586. 10.1111/1365-2435.12467

[fec13341-bib-0035] Mariotte, P. , Vandenberghe, C. , Kardol, P. , Hagedorn, F. , & Buttler, A. (2013). Subordinate plant species enhance community resistance against drought in semi‐natural grasslands. Journal of Ecology, 101, 763–773. 10.1111/1365-2745.12064

[fec13341-bib-0036] McGonigle, T. P. , Miller, M. H. , Evans, D. G. , Fairchild, G. L. , & Swan, J. A. (1990). A new method which gives an objective‐measure of colonization of roots by vesicular arbuscular mycorrhizal fungi. New Phytologist, 115, 495–501. 10.1111/j.1469-8137.1990.tb00476.x 33874272

[fec13341-bib-0037] Metcalfe, D. B. , Fisher, R. A. , & Wardle, D. A. (2011). Plant communities as drivers of soil respiration: Pathways, mechanisms, and significance for global change. Biogeosciences, 8, 2047–2061. 10.5194/bg-8-2047-2011

[fec13341-bib-0038] Metz, J. , von Oppen, J. , & Tielborger, K. (2015). Parental environmental effects due to contrasting watering adapt competitive ability, but not drought tolerance, in offspring of a semi‐arid annual Brassicaceae. Journal of Ecology, 103, 990–997. 10.1111/1365-2745.12411

[fec13341-bib-0039] Mousseau, T. A. , Uller, T. , Wapstra, E. , & Badyaev, A. V. (2009). Evolution of maternal effects: Past and present. Philosophical Transactions of the Royal Society B‐Biological Sciences, 364, 1035–1038. 10.1098/rstb.2008.0303 PMC2666690 19324608

[fec13341-bib-0040] Münzbergová, Z. , & Hadincová, V. (2017). Transgenerational plasticity as an important mechanism affecting response of clonal species to changing climate. Ecology and Evolution, 7, 5236–5247. 10.1002/ece3.3105 28770062 PMC5528211

[fec13341-bib-0041] R Core Team . (2015). R: A language and environment for statistical computing. Vienna, Austria: R Foundation for Statistical Computing.

[fec13341-bib-0042] Roach, D. A. , & Wulff, R. D. (1987). Maternal effects in plants. Annual Review of Ecology and Systematics, 18, 209–235. 10.1146/annurev.ecolsys.18.1.209

[fec13341-bib-0043] Rodwell, J. S. (1998). British plant communities. Cambridge, UK: Cambridge University Press.

[fec13341-bib-0044] Rottstock, T. , Kummer, V. , Fischer, M. , & Joshi, J. (2017). Rapid transgenerational effects in *Knautia arvensis* in response to plant community diversity. Journal of Ecology, 105, 714–725. 10.1111/1365-2745.12689

[fec13341-bib-0045] Shea, N. , Pen, I. , & Uller, T. (2011). Three epigenetic information channels and their different roles in evolution. Journal of Evolutionary Biology, 24, 1178–1187. 10.1111/j.1420-9101.2011.02235.x 21504495 PMC3116147

[fec13341-bib-0046] Smith, R. A. , Levine, T. R. , Lachlan, K. A. , & Fediuk, T. A. (2002). The high cost of complexity in experimental design and data analysis: Type I and type II error rates in multiway ANOVA. Human Communication Research, 28, 515–530. 10.1093/hcr/28.4.515

[fec13341-bib-0047] Steinauer, K. , Tilman, D. , Wragg, P. D. , Cesarz, S. , Cowles, J. M. , Pritsch, K. , … Eisenhauer, N. (2015). Plant diversity effects on soil microbial functions and enzymes are stronger than warming in a grassland experiment. Ecology, 96, 99–112. 10.1890/14-0088.1 26236895

[fec13341-bib-0048] Sultan, S. E. , Barton, K. , & Wilczek, A. M. (2009). Contrasting patterns of transgenerational plasticity in ecologically distinct congeners. Ecology, 90, 1831–1839. 10.1890/08-1064.1 19694132

[fec13341-bib-0049] Verhoeven, K. J. F. , & van Gurp, T. P. (2012). Transgenerational effects of stress exposure on offspring phenotypes in apomictic dandelion. PLoS ONE, 7, 1–8. 10.1371/journal.pone.0038605 PMC3377677 22723869

[fec13341-bib-0050] Vogel, A. , Eisenhauer, N. , Weigelt, A. , & Scherer‐Lorenzen, M. (2013). Plant diversity does not buffer drought effects on early‐stage litter mass loss rates and microbial properties. Global Change Biology, 19, 2795–2803. 10.1111/gcb.12225 23606531

[fec13341-bib-0051] Vogel, A. , Fester, T. , Eisenhauer, N. , Scherer‐Lorenzen, M. , Schmid, B. , Weisser, W. W. , & Weigelt, A. (2013). Separating drought effects from roof artifacts on ecosystem processes in a grassland drought experiment. PLoS ONE, 8, 1–10. 10.1371/journal.pone.0070997 PMC3731277 23936480

[fec13341-bib-0052] Walter, J. , Harter, D. E. V. , Beierkuhnlein, C. , & Jentsch, A. (2016). Transgenerational effects of extreme weather: Perennial plant offspring show modified germination, growth and stoichiometry. Journal of Ecology, 104, 1032–1040. 10.1111/1365-2745.12567

[fec13341-bib-0053] Weigelt, A. , Bol, R. , & Bardgett, R. D. (2005). Preferential uptake of soil nitrogen forms by grassland plant species. Oecologia, 142, 627–635. 10.1007/s00442-004-1765-2 15549402

[fec13341-bib-0054] Zuppinger‐Dingley, D. , Flynn, D. F. B. , De Deyn, G. B. , Petermann, J. S. , & Schmid, B. (2016). Plant selection and soil legacy enhance long‐term biodiversity effects. Ecology, 97, 918–928. 10.1890/15-0599.1 27220208

