# Supplementary material for: Drought soil legacy overrides maternal effects on plant growth
Source: Funct Ecol. 2019 Apr 29;33(8):1400–10. doi: 10.1111/1365-2435.13341 (PMC6767434; doi:10.1111/1365-2435.13341)
Supplement: Supplementary file 1 [file FEC-33-1400-s001.pdf]

Functional Ecology

BRITISH ECOLOGICAL SOCIETY

Drought soil legacy overrides maternal effects on plant growth

Jonathan R. De Long, Marina Semchenko, William J. Pritchard, Irene Cordero, Ellen L. Fry, Benjamin G. Jackson, Ksenia Kurnosova, Nicholas J. Ostle, David Johnson, Elizabeth M. Baggs and Richard D. Bardgett

Maternal plants can make changes to their seeds or the genetic expression of their offspring that allow the next generation to better cope with the same environmental conditions experienced by their mother through so-called maternal effects. In this way, plants can ensure their offspring are better equipped to survive harsh conditions, such as drought. In addition, climate events such as drought or even differences in plant community diversity can leave legacy effects in the soil that affect how future plants grow. However, interactions between maternal effects and soil legacies that could affect plant performance remain poorly understood. Here, we performed a glasshouse experiment using soil and seeds from a plant diversity and drought field experiment in the Yorkshire Dales National Park, England to test maternal, soil drought and diversity legacy effects, and their interactions, on offspring plant performance of two common grass species, Meadow Foxtail and Yorkshire Fog. Although maternal effects were detected, we found that drought soil legacy effects on plant growth were stronger than maternal effects. Drought soil legacy effects changed soil nutrient availability, microbial carbon allocation and enzyme activity, which

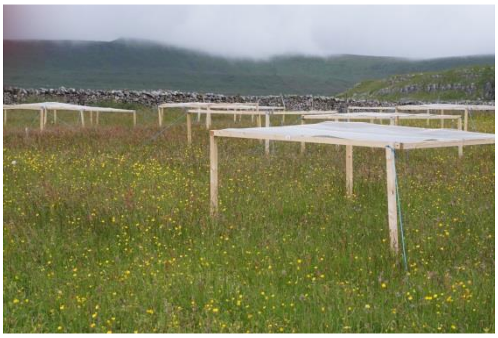

Drought shelters preventing rainfall from reaching subplots in a plant community diversity experiment located in Yorkshire Dales National Park, England. Seeds and soils were sampled from this field experiment to test for maternal effects and drought and plant diversity soil legacy effects. Photo credit: Jonathan R. De Long

may have affected plant growth. Further, plant tissue nutrient concentrations (i.e., carbon and nitrogen) and soil microbial responses to drought legacies varied between the two plant species and soils from high and low plant diversity treatments. However, these diversity effects did not affect how large plant roots or shoots grew. These findings demonstrate that maternal effects resulting from drought are likely to have minor impacts on plant performance compared to the effects of drought soil legacy effects. This suggests that soil drought legacy effects could change plant communities and the functions they control as extreme weather events become more frequent and intense with climate change.
