# Supplementary material for: Drought soil legacy overrides maternal effects on plant growth
Source: Funct Ecol. 2019 Apr 29;33(8):1400–10. doi: 10.1111/1365-2435.13341 (PMC6767434; doi:10.1111/1365-2435.13341)
Supplement: Supplementary file 2 [file FEC-33-1400-s002.docx]

## Supporting Information

TABLE S1. Plant species from different functional groups (i.e., grasses, forbs, legumes) were added to treatment plots as seeds and seedlings during the course of the experiment. Table originally printed in De Long et al. 2019.

|  |  |  |  | Seeds |  |  | Seedlings | |  |
| --- | --- | --- | --- | --- | --- | --- | --- | --- | --- |
| Species |  | Functional group |  | 2014^*^ | 2015^+^ |  | 2013^**^ | 2014^*^ | 2015^*^ |
| *Cynosurus cristatus* |  | Grass |  | X | X |  | X | X |  |
| *Dactylis glomerata* |  | Grass |  | X | X |  | X | X | X |
| *Festuca rubra* |  | Grass |  | X | X |  | X | X |  |
| *Poa trivialis* |  | Grass |  | X |  |  | X | X |  |
| *Briza media* |  | Grass |  | X | X |  | X | X | X |
| *Achillea millefolium* |  | Forb |  | X | X |  | X | X | X |
| *Geranium sylvaticum* |  | Forb |  | X | X |  | X | X | X |
| *Geum rivale* |  | Forb |  | X | X |  | X | X | X |
| *Leucanthemum vulgare* |  | Forb |  | X | X |  | X | X | X |
| *Plantago lanceolata* |  | Forb |  | X |  |  | X | X |  |
| *Prunella vulgaris* |  | Forb |  | X | X |  | X | X |  |
| *Hypochaeris radicata* |  | Forb |  | X | X |  | X | X |  |
| *Leontodon hispidus* |  | Forb |  | X | X |  | X | X |  |
| *Filipendula ulmaria* |  | Forb |  | X | X |  | X | X | X |
| *Centaurea nigra* |  | Forb |  | X |  |  | X | X |  |
| *Lathyrus pratensis* |  | Legume |  | X | X |  | X | X | X |
| *Lotus corniculatus* |  | Legume |  | X | X |  | X | X | X |
| *Trifolium pratense* |  | Legume |  | X | X |  | X | X |  |
| *Trifolium repens* |  | Legume |  | X |  |  | X | X |  |

^*^ Seeded in May

^**^ Planted in May and September

^+^ Seeded in March, April, and September

TABLE S2. Number of seeds from each functional group sown per plot in the different plant functional group treatments. See Table S1 for frequency of sowing and which species from each functional group were used. Table originally printed in De Long et al. 2019.

| 2014 |  |  |  |  |  |  |  |  |  |
| --- | --- | --- | --- | --- | --- | --- | --- | --- | --- |
|  |  | Functional group | |  |  |  |  |  |  |
| Treatment |  | Total forbs | Each forb species |  | Total legumes | Each legume species |  | Total grasses | Each grass species |
| Control |  | 0 | 0 |  | 0 | 0 |  | 0 | 0 |
| Forbs (F) |  | 64800 | 8100 |  | 0 | 0 |  | 0 | 0 |
| Legumes (L) |  | 0 | 0 |  | 64800 | 21600 |  | 0 | 0 |
| Grasses (G) |  | 0 | 0 |  | 0 | 0 |  | 64800 | 16200 |
| G+F |  | 32400 | 4050 |  | 0 | 0 |  | 32400 | 8100 |
| G+L |  | 0 | 0 |  | 32400 | 10800 |  | 32400 | 8100 |
| F+L |  | 32400 | 4050 |  | 32400 | 10800 |  | 0 | 0 |
| G+F+L |  | 21600 | 2700 |  | 21600 | 7200 |  | 21600 | 5400 |
| Total |  | 151200 | 18900 |  | 151200 | 50400 |  | 151200 | 37800 |

| 2015 |  |  |  |  |  |  |  |  |  |
| --- | --- | --- | --- | --- | --- | --- | --- | --- | --- |
|  |  | Functional group | |  |  |  |  |  |  |
| Treatment |  | Total forbs | Each forb species |  | Total legumes | Each legume species |  | Total grasses | Each grass species |
| Control |  | 0 | 0 |  | 0 | 0 |  | 0 | 0 |
| Forbs (F) |  | 97200 | 12150 |  | 0 | 0 |  | 0 | 0 |
| Legumes (L) |  | 0 | 0 |  | 97200 | 32400 |  | 0 | 0 |
| Grasses (G) |  | 0 | 0 |  | 0 | 0 |  | 97200 | 24300 |
| G+F |  | 48600 | 6075 |  | 0 | 0 |  | 48600 | 12150 |
| G+L |  | 0 | 0 |  | 48600 | 16200 |  | 48600 | 12150 |
| F+L |  | 48600 | 6075 |  | 48600 | 16200 |  | 0 | 0 |
| G+F+L |  | 32400 | 4050 |  | 32400 | 10800 |  | 32400 | 8100 |
| Total |  | 226800 | 28350 |  | 226800 | 75600 |  | 226800 | 56700 |

TABLE S3. Number of plugs of different species from each functional group sown per 6 x 6 m plot from the different plant functional group treatments. See Table S1 for species used and in which year each species was planted. Within each functional group, an equal number of each species was planted. Table originally printed in De Long et al. 2019.

| 2013, 2014 |  |  |  |  |
| --- | --- | --- | --- | --- |
|  |  | Functional group | |  |
| Treatment |  | Forbs | Legumes | Grasses |
| Control |  | 0 | 0 | 0 |
| Forbs (F) |  | 300 | 0 | 0 |
| Legumes (L) |  | 0 | 300 | 0 |
| Grasses (G) |  | 0 | 0 | 300 |
| G+F |  | 150 | 0 | 150 |
| G+L |  | 0 | 150 | 150 |
| F+L |  | 150 | 150 | 0 |
| G+F+L |  | 100 | 100 | 100 |
| Total |  | 3500 | 3500 | 3500 |

| 2015 |  |  |  |  |
| --- | --- | --- | --- | --- |
|  |  | Functional group | |  |
| Treatment |  | Forbs | Legumes | Grasses |
| Control |  | 0 | 0 | 0 |
| Forbs (F) |  | 100 | 0 | 0 |
| Legumes (L) |  | 0 | 100 | 0 |
| Grasses (G) |  | 0 | 0 | 100 |
| G+F |  | 50 | 0 | 50 |
| G+L |  | 0 | 50 | 50 |
| F+L |  | 50 | 50 | 0 |
| G+F+L |  | 33 | 33 | 33 |
| Total |  | 1150 | 1150 | 1150 |

SUPPORTING APPENDIX S1.

Microbial biomass: This involved extracting a non-fumigated 5 g (wet weight) subsample of soil with 25 mL of 0.5 M K_2_SO_4_ and another subsample fumigated with chloroform for 24 h, and measuring microbial C and N in each extract. Fumigation versus non-fumigation allows for a comparison of the C and N contained in dead versus living pools (i.e., contained with microbial cells) within the soil (Brookes, Landman, Pruden & Jenkinson 1985). “To account for baseline C and N mineralization, all values for microbial C were multiplied by a K_EC_ factor of 0.45 and all values of microbial N were multiplied by a K_EN_ factor of 0.54 (Brookes, Landman, Pruden & Jenkinson 1985). Samples were stored frozen (-18 ˚C) until analysis.

Soil extracellular enzyme activity: All activities were measured within 2 weeks after soil sampling. All activities were measured in triplicate and an average of these values was used to generate a single data point. β-Glucosidase (GLC), β-xylosidase (XYL), β-N-acetylglucosaminidase (NAG) and acid phosphatase (PHO) were measured photometrically (Jackson, Tyler & Millar 2013). A subsample of 3.75 g of sieved soil was suspended in 5 mL of sodium acetate buffer (50 mM, pH 5.0). A soil slurry of 150 μl was introduced into a 96-well deep well block and mixed with 150 μL of a saturating substrate solution: 20 mM pNP-β-glucopyranoside for GLC, 10 mM pNP-β-xylopyranoside for XYL, 5 mM pNP-β-N-acetylglucosaminide for NAG and 10 mM phosphatase substrate for PHO. Plates were incubated at 18 ºC for 0.5 h (PHO), 1.5 h (GLC) or 3.5 h (XYL and NAG) under continuous shaking. Blocks were centrifuged (2900 × *g*, 5 min), then 100 µL of the supernatant was pipetted into transparent 96-well plates and mixed with 200 µl of 50 mM NaOH solution. Absorbance was measured at 405 nm and compared with a calibration curve of known pNP concentration.

Phenoloxidase (POX) and peroxidase (PER) activities were measured photometrically according to Kaiser et al. (2010) with slight modifications. A subsample of 0.25 g of soil was suspended in 25 mL of sodium acetate buffer (50 mM, pH 5.0). A soil slurry of 0.4 mL was extracted under continuous stirring, transferred to a 96-well deep well block, mixed (1:1) with a 20 mM L-3,4-dihydroxyphenylalanin (L-DOPA) solution, shaken for 10 min and centrifuged (2900 × *g*, 5 min). A total of 250 µL of the supernatant was pipetted into transparent 96-well plates. For peroxidase activity, wells additionally received 10 µL of a 0.3 % H_2_O_2_ solution. Absorbance was measured at 450 nm at the starting time point and after 1.5 h (PER) or 20 h (POX) of incubation in the dark at 18 ºC. Enzyme activity was calculated from the difference in absorption between the two time-points divided by L-DOPA molar extinction coefficient (7.9 µmol^-1^) (DeForest 2009).

Urease (URE) and amino acid deaminase (DEA) were evaluated by the ammonia production according to Kandeler and Gerber (1988) with modifications. One g of soil was incubated with 500 µL of 80 mM urea solution (URE) or 15 mM L-arginine and glycine solution (DEA), at 18 ºC for 2 h (URE) or 3.5 h (DEA). After incubation, soils were extracted with 10/5 mL (URE/DEA) of 2 M KCl. Ammonia in extracts before and after incubation were evaluated with a Seal AA3 Segmented Flow Multi-chemistry analyser (Mequon, WI, USA) by the Berthelot reaction, which is a colorimetric reaction where ammonia, in the presence of salycilate and dichloroisocyanurate, is transformed into a green/blue coloured compound (Krom 1980).

TABLE S4A. Results of mixed effects models (*F* and *P* values) testing for the effects of maternal origin, drought legacy, diversity legacy, watering and species on plant (*Alopecurus pratensis* and *Holcus lanatus*) biomass and mycorrhizal colonisation; significant F- and *P*-values at *p* < 0.05 bolded. See Table S4B for degrees of freedom.

|  | Shoot biomass ^a^ | Root biomass ^a^ | Root to shoot ratio ^a^ | Arbuscule colonization ^b^ | Hyphae colonization ^b^ | Vesicle colonization ^b^ |
| --- | --- | --- | --- | --- | --- | --- |
|  | F-value (P) | F-value (P) | F-value (P) | F-value (P) | F-value (P) | F-value (P) |
| Maternal origin (M) | 0.1 (0.773) | 0.3 (0.585) | 0.4 (0.545) | 0.0 (0.876) | 0.1 (0.706) | 0.0 (0.965) |
| Drought legacy (L) | **6.2 (0.015)** | 1.6 (0.208) | 0.3 (0.605) | 1.7 (0.192) | 1.4 (0.246) | 3.4 (0.067) |
| Diversity legacy (D) | 1.1 (0.358) | 1.0 (0.350) | 0.0 (0.913) | 0.0 (0.880) | 1.4 (0.295) | 1.8 (0.224) |
| Watering (W) | **19.3 (<0.001)** | **5.3 (0.024)** | 0.1 (0.758) | 1.1 (0.290) | **7.0 (0.010)** | **8.7 (0.004)** |
| Species (S) | **226.1 (<0.001)** | **60.9 (<0.001)** | 3.3 (0.074) | **5.1 (0.026)** | **7.3 (0.008)** | **6.4 (0.013)** |
| M × L | **4.5 (0.037)** | 2.0 (0.157) | 0.0 (0.891) | 0.7 (0.412) | 0.2 (0.660) | 0.2(0.696) |
| M × D | **3.9 (0.050)** | 0.1 (0.780) | 2.3 (0.132) | **4.9 (0.029)** | **5.4 (0.022)** | 1.7 (0.199) |
| M × W | 0.3 (0.569) | 0.1 (0.711) | 0.4 (0.512) | 0.0 (0.886) | 0.0 (0.991) | 0.6 (0.448) |
| M × S | 0.1 (0.716) | 3.7 (0.058) | 2.8 (0.097) | 1.8 (0.182) | 3.4 (0.069) | 2.5 (0.119) |
| L × D | 0.2 (0.682) | 0.2 (0.690) | 0.5 (0.468) | 0.5 (0.484) | 0.1 (0.757) | 0.0 (0.848) |
| L × W | 0.1 (0.796) | 0.2 (0.632) | 0.5 (0.488) | 0.4 (0.547) | 1.5 (0.219) | 0.3 (0.564) |
| L × S | 0.2 (0.655) | 0.0 (0.867) | 0.0 (0.861) | 1.2 (0.285) | 0.1 (0.805) | 0.1 (0.748) |
| D × W | **5.2 (0.025)** | 0.6 (0.433) | 0.9 (0.337) | 0.0 (0.999) | 0.0 (0.911) | 0.1 (0.802) |
| D × S | 2.5 (0.114) | 0.8 (0.383) | 0.0 (0.879) | 0.0 (0.890) | 0.6 (0.443) | 1.2 (0.272) |
| W × S | **9.3 (0.003)** | 0.1 (0.721) | **4.7 (0.033)** | 0.0 (0.931) | 0.1 (0.801) | 0.0 (0.901) |
| M × L × D | 1.5 (0.222) | 0.0 (0.905) | 0.6 (0.444) | 0.1 (0.750) | 3.1 (0.082) | 2.6 (0.108) |
| M × L × W | 0.4 (0.548) | 0.6 (0.443) | 0.0 (0.954) | 0.0 (0.850) | 0.7 (0.407) | 0.0 (0.963) |
| M × L × S | 1.3 (0.264) | 1.1 (0.299) | 0.3 (0.595) | 0.4 (0.530) | 0.0 (0.850) | 0.1 (0.708) |
| M × D × W | 0.0 (0.991) | 1.9 (0.171) | 0.7 (0.407) | 0.1 (0.778) | 1.1 (0.288) | 0.5 (0.504) |
| M × D × S | 2.6 (0.112) | 0.0 (0.855) | 0.6 (0.457) | 0.4 (0.550) | 0.4 (0.544) | 0.1 (0.795) |
| M × W × S | 0.0 (0.841) | 0.3 (0.597) | 0.1 (0.705) | 1.6 (0.212) | 0.0 (0.833) | 1.1 (0.296) |
| L × D × W | 0.2 (0.622) | 0.1 (0.803) | 0.1 (0.829) | 0.1 (0.742) | 0.0 (0.897) | 0.0 (0.853) |
| L × D × S | 0.1 (0.775) | 2.8 (0.099) | 2.8 (0.099) | 3.6 (0.059) | 3.4 (0.068) | **4.4 (0.038)** |
| L × W × S | 1.9 (0.171) | 0.2 (0.668) | 2.7 (0.101) | 0.1 (0.724) | 1.9 (0.168) | 1.2 (0.276) |
| D × W × S | 1.0 (0.311) | 0.1 (0.778) | 2.0 (0.161) | 0.6 (0.452) | 0.1 (0.805) | 0.4 (0.535) |

^a^ Data ln(x) transformed before analysis ^b^ Data arcsin(sqrt(x)) transformed before analysis

TABLE S4B. Table containing the degrees of freedom, denominator degrees of freedom corresponding to the analyses shown in Table S4A.

|  | Shoot biomass | Root biomass | Root to shoot ratio | Arbuscule colonization | Hyphae colonization | Vesicle colonization |
| --- | --- | --- | --- | --- | --- | --- |
| Maternal origin (M) | 1,96.33 | 1,99.429 | 1,100.162 | 1,110.48 | 1,98.337 | 1,99.311 |
| Drought legacy (L) | 1,96.684 | 1,98.48 | 1,100.309 | 1,108.85 | 1,96.623 | 1,96.18 |
| Diversity legacy (D) | 1,4.14 | 1,8.984 | 1,107.161 | 1,111.93 | 1,4.061 | 1,6.924 |
| Watering (W) | 1,96.043 | 1,98.127 | 1,100.253 | 1,108.94 | 1,96.098 | 1,95.901 |
| Species (S) | 1,96.876 | 1,99.725 | 1,100.326 | 1,109.66 | 1,98.53 | 1,99.334 |
| M × L | 1,96.215 | 1,98.366 | 1,100.433 | 1,109.64 | 1,96.414 | 1,96.425 |
| M × D | 1,105.778 | 1,106.528 | 1,107.889 | 1,110.91 | 1,106.312 | 1,107.357 |
| M × W | 1,96.205 | 1,98.214 | 1,100.327 | 1,109.25 | 1,96.346 | 1,96.147 |
| M × S | 1,95.729 | 1,98.284 | 1,99.779 | 1,109.34 | 1,96.417 | 1,96.727 |
| L × D | 1,100.866 | 1,100.662 | 1,102.876 | 1,109.02 | 1,100.536 | 1,98.835 |
| L × W | 1,95.489 | 1,97.969 | 1,99.808 | 1,109.17 | 1,96.091 | 1,96.294 |
| L × S | 1,97.082 | 1,98.655 | 1,100.533 | 1,108.85 | 1,97.028 | 1,96.426 |
| D × W | 1,99.947 | 1,100.25 | 1,102.567 | 1,109.12 | 1,99.635 | 1,98.34 |
| D × S | 1,100.986 | 1,102.281 | 1,103.421 | 1,111.32 | 1,101.804 | 1,102.302 |
| W × S | 1,95.556 | 1,97.768 | 1,100.245 | 1,109.37 | 1,95.616 | 1,95.5 |
| M × L × D | 1,104.74 | 1,104.679 | 1,106.064 | 1,108.76 | 1,104.054 | 1,103.896 |
| M × L × W | 1,94.991 | 1,97.414 | 1,99.581 | 1,108.82 | 1,95.182 | 1,95.071 |
| M × L × S | 1,96.543 | 1,98.484 | 1,100.641 | 1,109.67 | 1,96.796 | 1,96.63 |
| M × D × W | 1,104.599 | 1,104.275 | 1,106.644 | 1,108.99 | 1,103.834 | 1,103.094 |
| M × D × S | 1,100.43 | 1,101.002 | 1,102.734 | 1,109.93 | 1,100.697 | 1,100.062 |
| M × W × S | 1,95.711 | 1,97.896 | 1,100.285 | 1,109.53 | 1,95.907 | 1,95.798 |
| L × D × W | 1,104.117 | 1,103.767 | 1,106.244 | 1,109.3 | 1,103.375 | 1,102.647 |
| L × D × S | 1,101.317 | 1,101.185 | 1,103.404 | 1,109.05 | 1,100.84 | 1,99.45 |
| L × W × S | 1,95.507 | 1,97.998 | 1,99.907 | 1,109.16 | 1,96.058 | 1,96.282 |
| D × W × S | 1,98.029 | 1,99.189 | 1,101.381 | 1,108.58 | 1,97.743 | 1,96.933 |

TABLE S5A. Results of mixed effects models (*F* and *P* values) testing for the effects of maternal origin, drought legacy, diversity legacy, watering and species on plant (*Alopecurus pratensis* and *Holcus lanatus*) nutrient concentrations; signficant F- and *P*-values at *p* < 0.05 bolded. See Table S5B for degrees of freedom.

|  | Shoot %C ^b^ | Shoot %N ^b^ | Shoot C:N ^a^ | Shoot ^15^N enrichment ^b^ | Root %C ^b^ | Root %N ^b^ | Root C:N ^a^ | Root ^15^N enrichment ^b^ |
| --- | --- | --- | --- | --- | --- | --- | --- | --- |
|  | F-value (P) | F-value (P) | F-value (P) | F-value (P) | F-value (P) | F-value (P) | F-value (P) | F-value (P) |
| Maternal origin (M) | 0.1 (0.765) | 0.0 (0.911) | 0.0 (0.957) | 0.3 (0.595) | 0.0 (0.985) | 0.4 (0.558) | 0.2 (0.664) | 0.0 (0.888) |
| Drought legacy (L) | 3.4 (0.070) | **16.4 (<0.001)** | **11.3 (<0.001)** | **20.7 (<0.001)** | 1.1 (0.309) | **9.6 (0.003)** | 2.1 (0.153) | **18.3 (<0.001)** |
| Diversity legacy (D) | 0.2 (0.644) | 0.4 (0.569) | 0.4 (0.531) | 0.5 (0.488) | 0.5 (0.483) | 0.6 (0.456) | 0.0 (0.980) | 0.4 (0.544) |
| Watering (W) | **4.6 (0.035)** | **17.2 (<0.001)** | **12.6 (0.001)** | 0.0 (0.981) | 0.0 (0.947) | 0.3 (0.565) | 0.2 (0.622) | 1.5 (0.226) |
| Species (S) | **53.0 (<0.001)** | **434.3 (<0.001)** | **351.9 (<0.001)** | **66.6 (<0.001)** | 0.1 (0.707) | **37.9 (<0.001)** | **16.4 (<0.001)** | 2.9 (0.090) |
| M × L | 1.5 (0.225) | 2.0 (0.156) | 1.2 (0.274) | 1.0 (0.331) | 0.2 (0.681) | 0.2 (0.673) | 0.3 (0.560) | 0.2 (0.667) |
| M × D | 2.7 (0.101) | 0.0 (0.872) | 0.1 (0.813) | 0.3 (0.564) | 0.8 (0.387) | 0.3 (0.562) | 0.0 (0.992) | 0.0 (0.947) |
| M × W | 0.9 (0.334) | 1.0 (0.329) | 0.9 (0.339) | 2.8 (0.098) | 0.1 (0.769) | 1.5 (0.232) | 0.8 (0.381) | 3.5 (0.065) |
| M × S | **4.0 (0.048)** | 1.1 (0.291) | 1.9 (0.170) | 0.7 (0.403) | 0.2 (0.658) | 0.1 (0.720) | 0.0 (0.966) | 1.0 (0.326) |
| L × D | 0.0 (0.841) | 1.9 (0.171) | 2.4 (0.123) | 2.2 (0.141) | 1.0 (0.325) | 1.5 (0.217) | 2.4 (0.122) | 0.9 (0.333) |
| L × W | 0.0 (0.838) | 0.6 (0.439) | 0.7 (0.392) | 0.4 (0.516) | 0.1 (0.725) | 2.2 (0.146) | 0.4 (0.526) | 0.5 (0.499) |
| L × S | 0.9 (0.349) | 1.7 (0.194) | 0.4 (0.528) | 1.7 (0.190) | 0.7 (0.401) | 1.1 (0.287) | 1.9 (0.171) | 1.8 (0.183) |
| D × W | 0.3 (0.599) | 1.4 (0.234) | 1.5 (0.230) | 2.8 (0.097) | 0.2 (0.657) | 0.0 (0.847) | 0.0 (0.917) | 0.7 (0.394) |
| D × S | 1.2 (0.283) | 0.6 (0.427) | 0.9 (0.339) | 0.5 (0.496) | 0.7 (0.391) | 2.0 (0.156) | 2.4 (0.122) | 0.0 (0.850) |
| W × S | 0.5 (0.463) | 0.3 (0.589) | 0.0 (0.923) | 0.0 (0.938) | 1.7 (0.195) | 0.0 (0.893) | 0.6 (0.461) | 0.0 (0.966) |
| M × L × D | 0.2 (0.651) | 0.7 (0.416) | 1.1 (0.300) | 0.1 (0.819) | 0.8 (0.382) | 0.6 (0.432) | 0.0 (0.871) | 0.5 (0.464) |
| M × L × W | 1.2 (0.278) | 0.9 (0.334) | 0.7 (0.396) | 0.3 (0.598) | 0.0 (1.000) | 0.9 (0.345) | 0.5 (0.475) | 0.0 (0.846) |
| M × L × S | **4.2 (0.042)** | 0.4 (0.534) | 0.8 (0.378) | 2.4 (0.122) | 0.0 (0.996) | 2.9 (0.093) | 1.7 (0.201) | 0.2 (0.640) |
| M × D × W | **4.0 (0.048)** | 0.3 (0.595) | 0.0 (0.976) | 1.6 (0.208) | 1.4 (0.244) | 0.6 (0.457) | 0.3 (0.579) | 0.2 (0.639) |
| M × D × S | 0.1 (0.710) | 0.1 (0.805) | 0.0 (0.902) | 0.2 (0.685) | 3.5 (0.064) | 2.1 (0.150) | 0.3 (0.611) | 0.3 (0.602) |
| M × W × S | 0.0 (0.972) | 1.7 (0.191) | 1.0 (0.316) | 0.6 (0.426) | 0.0 (0.843) | 0.9 (0.351) | 0.3 (0.579) | 0.1 (0.726) |
| L × D × W | 0.2 (0.687) | 0.0 (0.950) | 0.0 (0.992) | 0.1 (0.825) | 0.2 (0.679) | 0.7 (0.405) | 0.1 (0.757) | 0.0 (0.857) |
| L × D × S | 0.2 (0.651) | 3.7 (0.056) | **4.9 (0.030)** | 0.1 (0.784) | 0.1 (0.783) | 2.6 (0.114) | 1.9 (0.172) | 0.9 (0.339) |
| L × W × S | 0.5 (0.464) | 2.4 (0.123) | 2.6 (0.112) | 0.8 (0.376) | 0.7 (0.409) | 0.1 (0.738) | 0.3 (0.611) | 0.2 (0.704) |
| D × W × S | 0.0 (0.861) | 0.0 (0.955) | 0.0 (0.943) | 0.4 (0.552) | 2.3 (0.129) | 0.4 (0.542) | 3.1 (0.082) | 0.1 (0.736) |

carbon, (C), nitrogen (N) ^a^ Data ln(x) transformed before analysis ^b^ Data arcsin(sqrt(x)) transformed before analysis

TABLE S5B. Table containing the degrees of freedom, denominator degrees of freedom corresponding to the analyses shown in Table S5A.

|  | Shoot %C | Shoot %N | Shoot C:N | Shoot ^15^N enrichment | Root %C | Root %N | Root C:N | Root ^15^N enrichment |
| --- | --- | --- | --- | --- | --- | --- | --- | --- |
| Maternal origin (M) | 1,101.789 | 1,102.28 | 1,103.028 | 1,99.855 | 1,112 | 1,100.574 | 1,103.916 | 1,96.707 |
| Drought legacy (L) | 1,99.688 | 1,101.23 | 1,101.985 | 1,98.885 | 1,112 | 1,99.43 | 1,102.421 | 1,94.647 |
| Diversity legacy (D) | 1,8.539 | 1,6.76 | 1,6.941 | 1,9.07 | 1,112 | 1,7.699 | 1,6.831 | 1,3.88 |
| Watering (W) | 1,99.542 | 1,100.83 | 1,101.622 | 1,99.037 | 1,112 | 1,99.061 | 1,102.131 | 1,94.756 |
| Species (S) | 1,101.874 | 1,102.4 | 1,103.143 | 1,100.056 | 1,112 | 1,100.806 | 1,103.903 | 1,97.362 |
| M × L | 1,99.895 | 1,101.11 | 1,101.899 | 1,98.618 | 1,112 | 1,99.337 | 1,102.537 | 1,95.113 |
| M × D | 1,107.489 | 1,102.28 | 1,103.04 | 1,105.877 | 1,112 | 1,106.549 | 1,105.965 | 1,103.891 |
| M × W | 1,99.66 | 1,101.07 | 1,101.837 | 1,99.099 | 1,112 | 1,99.202 | 1,102.405 | 1,94.376 |
| M × S | 1,100.15 | 1,101.01 | 1,101.821 | 1,98.798 | 1,112 | 1,99.273 | 1,102.563 | 1,94.85 |
| L × D | 1,101.041 | 1,103.45 | 1,103.969 | 1,102.875 | 1,112 | 1,101.833 | 1,104.372 | 1,97.436 |
| L × W | 1,99.792 | 1,100.79 | 1,101.615 | 1,98.841 | 1,112 | 1,98.974 | 1,102.486 | 1,95.232 |
| L × S | 1,99.79 | 1,101.54 | 1,102.267 | 1,99.955 | 1,112 | 1,99.659 | 1,102.696 | 1,94.911 |
| D × W | 1,100.895 | 1,103.13 | 1,103.701 | 1,101.977 | 1,112 | 1,101.367 | 1,104.13 | 1,96.157 |
| D × S | 1,103.904 | 1,105.05 | 1,105.675 | 1,103.53 | 1,112 | 1,103.507 | 1,106.487 | 1,99.177 |
| W × S | 1,99.222 | 1,100.54 | 1,101.344 | 1,98.271 | 1,112 | 1,98.713 | 1,101.892 | 1,94.565 |
| M × L × D | 1,105.086 | 1,100.91 | 1,101.689 | 1,105.009 | 1,112 | 1,104.718 | 1,104.231 | 1,102.418 |
| M × L × W | 1,98.925 | 1,100.15 | 1,100.981 | 1,98.358 | 1,112 | 1,98.33 | 1,101.574 | 1,93.954 |
| M × L × S | 1,99.952 | 1,101.44 | 1,102.198 | 1,99.701 | 1,112 | 1,99.527 | 1,102.842 | 1,95.233 |
| M × D × W | 1,104.354 | 1,101.71 | 1,102.31 | 1,104.869 | 1,112 | 1,104.405 | 1,104.016 | 1,101.335 |
| M × D × S | 1,102.206 | 1,103.97 | 1,104.573 | 1,103.065 | 1,112 | 1,102.238 | 1,105.467 | 1,97.709 |
| M × W × S | 1,99.376 | 1,100.77 | 1,101.567 | 1,98.573 | 1,112 | 1,98.887 | 1,102.21 | 1,93.569 |
| L × D × W | 1,104.208 | 1,103.13 | 1,103.662 | 1,104.894 | 1,112 | 1,104.181 | 1,105.091 | 1,100.838 |
| L × D × S | 1,101.674 | 1,103.55 | 1,104.095 | 1,102.687 | 1,112 | 1,102.251 | 1,104.608 | 1,98.5 |
| L × W × S | 1,99.796 | 1,100.74 | 1,101.577 | 1,98.44 | 1,112 | 1,98.985 | 1,102.449 | 1,95.321 |
| D × W × S | 1,100.133 | 1,101.96 | 1,102.648 | 1,99.943 | 1,112 | 1,100.181 | 1,103.044 | 1,95.588 |

TABLE S6A. Results of mixed effects models (*F* and *P* values) testing for the effects of maternal origin, drought legacy, diversity legacy, watering and species on soil abiotic properties after plants (*Alopecurus pratensis* and *Holcus lanatus*) were harvested; significant F- and *P*-values at *p* < 0.05 bolded. See Table S6B for degrees of freedom.

|  | TIN ^a^ | Microbial C ^a^ | Microbial N ^a^ | Microbial C:N ^a^ |
| --- | --- | --- | --- | --- |
|  | F-value (P) | F-value (P) | F-value (P) | F-value (P) |
| Maternal origin (M) | 0.1 (0.741) | 0.6 (0.424) | 0.0 (0.901) | 0.9 (0.350) |
| Drought legacy (L) | **8.3 (0.005)** | **6.1 (0.015)** | 0.1 (0.743) | **8.5 (0.004)** |
| Diversity legacy (D) | 0.0 (0.889) | 0.1 (0.825) | 0.4 (0.545) | 1.8 (0.245) |
| Watering (W) | **9.8 (0.002)** | 0.7 (0.415) | 3.9 (0.052) | 3.8 (0.053) |
| Species (S) | **93.9 (<0.001)** | 1.7 (0.196) | 2.4 (0.124) | 0.4 (0.523) |
| M × L | 1.4 (0.244) | 0.0 (0.884) | 0.2 (0.652) | 0.7 (0.416) |
| M × D | 0.2 (0.680) | 1.1 (0.306) | 0.0 (0.849) | 1.5 (0.228) |
| M × W | 3.1 (0.080) | 0.0 (0.926) | 0.6 (0.461) | 1.7 (0.199) |
| M × S | 1.4 (0.241) | 3.0 (0.086) | 2.0 (0.161) | 0.0 (0.846) |
| L × D | 0.8 (0.384) | 1.5 (0.230) | 1.5 (0.230) | 0.0 (0.854) |
| L × W | **4.5 (0.037)** | 0.4 (0.536) | 0.0 (0.950) | 0.7 (0.414) |
| L × S | 0.2 (0.639) | 0.0 (0.836) | 0.1 (0.754) | 0.0 (0.841) |
| D × W | **4.1 (0.045)** | 0.2 (0.662) | 0.2 (0.681) | 0.0 (0.980) |
| D × S | 0.2 (0.694) | 0.0 (0.853) | 0.6 (0.453) | 0.7 (0.394) |
| W × S | 0.5 (0.488) | 0.0 (0.867) | 1.6 (0.215) | 2.8 (0.095) |
| M × L × D | 0.9 (0.351) | 0.1 (0.735) | 1.3 (0.251) | 1.4 (0.239) |
| M × L × W | 0.7 (0.394) | 0.4 (0.530) | 2.2 (0.142) | 2.0 (0.165) |
| M × L × S | 0.2 (0.682) | 0.7 (0.409) | 0.2 (0.645) | 0.1 (0.730) |
| M × D × W | 0.6 (0.426) | 1.2 (0.276) | 0.1 (0.779) | 1.0 (0.310) |
| M × D × S | **4.6 (0.035)** | 0.4 (0.529) | 0.4 (0.552) | 0.0 (0.991) |
| M × W × S | 2.3 (0.131) | 0.0 (0.834) | 0.0 (0.906) | 0.0 (0.902) |
| L × D × W | 1.1 (0.289) | 0.5 (0.490) | 0.6 (0.443) | **4.3 (0.041)** |
| L × D × S | 0.9 (0.359) | 1.5 (0.226) | 0.5 (0.501) | **7.5 (0.008)** |
| L × W × S | 0.2 (0.655) | 1.2 (0.272) | 0.2 (0.688) | **4.9 (0.030)** |
| D × W × S | 0.2 (0.684) | 0.6 (0.439) | 1.0 (0.329) | 0.1 (0.722) |

C (carbon), N (nitrogen), total inorganic nitrogen (TIN)

^a^ Data ln(x) transformed before analysis

TABLE S6B. Table containing the degrees of freedom, denominator degrees of freedom corresponding to the analyses shown in Table S6A.

|  | TIN | Microbial C | Microbial N | Microbial C:N |
| --- | --- | --- | --- | --- |
| Maternal origin (M) | 1,96.688 | 1,96.971 | 1,96.722 | 1,97.898 |
| Drought legacy (L) | 1,95.225 | 1,96.617 | 1,96.398 | 1,96.411 |
| Diversity legacy (D) | 1,4.204 | 1,4.351 | 1,4.204 | 1,4.097 |
| Watering (W) | 1,95.251 | 1,96.355 | 1,96.193 | 1,96.18 |
| Species (S) | 1,96.836 | 1,97.342 | 1,97.022 | 1,98.224 |
| M × L | 1,94.896 | 1,96.389 | 1,96.252 | 1,96.306 |
| M × D | 1,101.039 | 1,101.027 | 1,100.18 | 1,101.157 |
| M × W | 1,95.401 | 1,96.324 | 1,96.17 | 1,96.153 |
| M × S | 1,95.145 | 1,96.476 | 1,96.277 | 1,96.689 |
| L × D | 1,96.175 | 1,97.41 | 1,97.059 | 1,96.827 |
| L × W | 1,94.821 | 1,96.241 | 1,96.065 | 1,96.246 |
| L × S | 1,95.562 | 1,96.648 | 1,96.415 | 1,96.394 |
| D × W | 1,95.719 | 1,97.252 | 1,96.958 | 1,96.771 |
| D × S | 1,98.019 | 1,98.309 | 1,98.005 | 1,99.183 |
| W × S | 1,94.364 | 1,96.002 | 1,95.916 | 1,95.69 |
| M × L × D | 1,99.816 | 1,100.323 | 1,99.548 | 1,100.037 |
| M × L × W | 1,94.417 | 1,95.98 | 1,95.854 | 1,95.742 |
| M × L × S | 1,95.338 | 1,96.365 | 1,96.224 | 1,96.222 |
| M × D × W | 1,99.788 | 1,99.918 | 1,99.286 | 1,99.366 |
| M × D × S | 1,96.465 | 1,97.595 | 1,97.259 | 1,97.585 |
| M × W × S | 1,94.387 | 1,96.046 | 1,95.948 | 1,95.757 |
| L × D × W | 1,98.435 | 1,99.553 | 1,98.999 | 1,99.032 |
| L × D × S | 1,96.677 | 1,97.762 | 1,97.389 | 1,97.299 |
| L × W × S | 1,95.319 | 1,96.256 | 1,96.082 | 1,96.238 |
| D × W × S | 1,95.544 | 1,96.898 | 1,96.635 | 1,96.517 |

TABLE S7. Results of mixed effects models (*F* and *P* values) testing for the effects of maternal origin, drought legacy, diversity legacy, watering and species on soil enzymes after plants (*Alopecurus pratensis* and *Holcus lanatus*) were harvested; significant F- and *P*-values at *p* < 0.05 bolded. See Table S7B for degrees of freedom.

|  | DEA ^a^ | URE ^a^ | GLC ^a^ | XYL ^a^ | NAG ^a^ | PHO ^a^ | PER ^a^ | POX ^a^ |
| --- | --- | --- | --- | --- | --- | --- | --- | --- |
|  | F-value (P) | F-value (P) | F-value (P) | F-value (P) | F-value (P) | F-value (P) | F-value (P) | F-value (P) |
| Maternal origin (M) | 0.2 (0.656) | 0.1 (0.807) | 1.2 (0.283) | 0.3 (0.569) | 0.4 (0.535) | 1.9 (0.167) | 2.1 (0.147) | 2.1 (0.150) |
| Drought legacy (L) | 2.7 (0.106) | **6.4 (0.013)** | 0.3 (0.592) | 0.5 (0.465) | 1.7 (0.200) | 1.6 (0.206) | 2.3 (0.135) | 0.2 (0.633) |
| Diversity legacy (D) | 2.5 (0.154) | 0.8 (0.414) | 2.5 (0.154) | 0.2 (0.622) | 2.1 (0.221) | 1.5 (0.283) | 0.9 (0.395) | 2.4 (0.195) |
| Watering (W) | 0.2 (0.667) | **4.4 (0.039)** | **21.3 (<0.001)** | **22.8 (<0.001)** | **24.6 (<0.001)** | **13.4 (<0.001)** | 0.0 (0.985) | 0.0 (0.848) |
| Species (S) | 1.2 (0.279) | **7.6 (0.007)** | 1.6 (0.215) | 3.2 (0.075) | 3.5 (0.065) | **5.8 (0.018)** | 0.3 (0.612) | 1.7(0.202) |
| M × L | 0.4 (0.508) | 0.5 (0.464) | 0.3 (0.590) | 0.0 (0.980) | 0.6 (0.439) | 0.2 (0.691) | 0.0 (0.845) | 0.0 (0.967) |
| M × D | 0.7 (0.415) | 0.3 (0.600) | 0.2 (0.697) | 0.8 (0.373) | 1.0 (0.326) | 0.0 (0.951) | 0.2 (0.647) | 0.0 (0.924) |
| M × W | 0.0 (0.922) | 0.5 (0.471) | 0.1 (0.756) | 0.3 (0.621) | 0.2 (0.642) | 0.9 (0.343) | 0.2(0.686) | 1.2 (0.269) |
| M × S | 0.3 (0.598) | 1.0 (0.316) | 0.0 (0.888) | 0.3 (0.571) | 0.0 (0.961) | 0.2 (0.628) | 0.3 (0.617) | 0.7 (0.408) |
| L × D | **5.1 (0.027)** | **11.5 (0.001)** | 1.6 (0.206) | 0.7 (0.411) | 2.0 (0.164) | 0.5 (0.480) | 2.5 (0.116) | 0.2 (0.661) |
| L × W | 0.0 (0.859) | 0.0 (0.896) | 2.3 (0.134) | 1.0 (0.314) | 1.5 (0.219) | 0.7 (0.396) | 0.0 (0.838) | 1.2 (0.277) |
| L × S | 0.5 (0.485) | 0.0 (0.891) | 2.0 (0.163) | 0.0 (0.878) | 0.1 (0.792) | 0.0 (0.881) | 0.4 (0.548) | 0.1 (0.758) |
| D × W | 3.9 (0.051) | 0.0 (0.929) | 0.8 (0.384) | 0.1 (0.777) | 0.1 (0.779) | 0.0 (0.862) | 0.1 (0.767) | 0.2 (0.625) |
| D × S | 2.1 (0.147) | 0.0 (0.912) | 0.6 (0.453) | 1.4 (0.241) | 0.0 (0.917) | 0.0 (0.842) | 1.2 (0.270) | 0.0 (0.852) |
| W × S | 0.2 (0.703) | 0.0 (0.922) | 1.3 (0.251) | 0.2 (0.659) | 1.1 (0.309) | 0.1 (0.831) | 0.6 (0.435) | 0.1 (0.820) |
| M × L × D | 0.0 (0.908) | 0.2 (0.669) | 0.2 (0.687) | 2.1 (0.155) | 0.1 (0.751) | 0.8 (0.385) | 0.1 (0.725) | 0.1 (0.745) |
| M × L × W | 0.3 (0.618) | 0.1 (0.807) | 1.2 (0.280) | 1.0 (0.323) | 0.8 (0.375) | 2.3 (0.133) | 2.4 (0.126) | 0.1 (0.810) |
| M × L × S | 1.1 (0.309) | 1.2 (0.284) | 1.7 (0.195) | 0.7 (0.421) | 1.7 (0.194) | **5.3 (0.024)** | 0.2 (0.663) | 0.5 (0.472) |
| M × D × W | 3.0 (0.087) | 0.1 (0.727) | 2.2 (0.140) | 0.1 (0.756) | 0.3 (0.568) | 1.1 (0.299) | 1.0 (0.319) | **4.6 (0.034)** |
| M × D × S | 0.3 (0.580) | 0.3 (0.603) | 0.0 (0.943) | 1.8 (0.184) | 0.6 (0.442) | 0.0 (0.870) | 0.0 (0.873) | 0.6 (0.461) |
| M × W × S | 0.3 (0.610) | 0.1 (0.787) | 0.1 (0.763) | 0.1 (0.781) | 0.0 (0.950) | 0.3 (0.597) | 0.0 (0.964) | 0.1 (0.785) |
| L × D × W | 0.7 (0.413) | 0.0 (0.896) | **4.4 (0.038)** | 2.3 (0.130) | 1.5 (0.218) | 2.5 (0.116) | 2.4 (0.123) | 1.2 (0.272) |
| L × D × S | 0.0 (0.963) | 0.1 (0.734) | **3.9 (0.050)** | 1.3 (0.260) | 0.0 (0.923) | 0.2 (0.669) | 1.1 (0.289) | 0.02 (0.892) |
| L × W × S | 0.4 (0.553) | 2.5 (0.120) | 1.0 (0.322) | 1.0 (0.309) | 0.0 (0.938) | 0.1 (0.776) | **14.5 (<0.001)** | 3.8 (0.056) |
| D × W × S | 0.6 (0.428) | 0.3 (0.565) | 0.1 (0.797) | 0.7 (0.408) | 0.1 (0.805) | 0.0 (0.927) | 0.0 (0.906) | 0.4 (0.541) |

Amino acid deaminase (DEA), Glucosidase (GLC), N-acetylglucosaminidase (NAG), peroxidase (PER), phenoloxidase (POX), phosphatase (PHO), urease (URE), xylosidase (XYL)

^a^ Data ln(x) transformed before analysis

TABLE S7B. Table containing the degrees of freedom, denominator degrees of freedom corresponding to the analyses shown in Table S7A.

|  | DEA | URE | GLC | XYL | NAG | PHO | PER | POX |
| --- | --- | --- | --- | --- | --- | --- | --- | --- |
| Maternal origin (M) | 1,96.895 | 1,97.06 | 1,97.325 | 1,96.292 | 1,96.754 | 1,96.819 | 1,96.886 | 1,96.438 |
| Drought legacy (L) | 1,95.837 | 1,97.594 | 1,96.784 | 1,96.107 | 1,96.231 | 1,96.734 | 1,96.405 | 1,96.098 |
| Diversity legacy (D) | 1,8.121 | 1,4.157 | 1,8.191 | 1,8.357 | 1,4.217 | 1,4.101 | 1,4.086 | 1,4.122 |
| Watering (W) | 1,95.629 | 1,97.137 | 1,96.628 | 1,95.958 | 1,96.017 | 1,96.472 | 1,96.144 | 1,95.818 |
| Species (S) | 1,97.197 | 1,97.61 | 1,97.577 | 1,96.523 | 1,97.073 | 1,97.187 | 1,97.233 | 1,96.839 |
| M × L | 1,95.824 | 1,97.21 | 1,96.747 | 1,96.037 | 1,96.091 | 1,96.523 | 1,96.279 | 1,95.873 |
| M × D | 1,101.589 | 1,104.68 | 1,100.575 | 1,99.336 | 1,100.359 | 1,101.516 | 1,102.317 | 1,101.307 |
| M × W | 1,95.638 | 1,97.175 | 1,96.618 | 1,95.941 | 1,95.995 | 1,96.45 | 1,96.144 | 1,95.793 |
| M × S | 1,95.86 | 1,96.871 | 1,96.732 | 1,95.968 | 1,96.16 | 1,96.468 | 1,96.203 | 1,95.902 |
| L × D | 1,96.808 | 1,100.132 | 1,97.41 | 1,96.706 | 1,96.909 | 1,97.772 | 1,97.662 | 1,97.136 |
| L × W | 1,95.479 | 1,96.693 | 1,96.479 | 1,95.804 | 1,95.9 | 1,96.28 | 1,95.927 | 1,95.656 |
| L × S | 1,95.864 | 1,97.799 | 1,96.79 | 1,96.117 | 1,96.247 | 1,96.785 | 1,96.467 | 1,96.148 |
| D × W | 1,96.719 | 1,99.603 | 1,97.366 | 1,96.647 | 1,96.809 | 1,97.588 | 1,97.474 | 1,96.945 |
| D × S | 1,98.942 | 1,100.294 | 1,98.794 | 1,97.57 | 1,98.122 | 1,98.499 | 1,99.032 | 1,98.129 |
| W × S | 1,95.304 | 1,96.733 | 1,96.394 | 1,95.769 | 1,95.703 | 1,96.164 | 1,95.821 | 1,95.452 |
| M × L × D | 1,100.209 | 1,103.951 | 1,99.789 | 1,98.782 | 1,99.597 | 1,100.794 | 1,101.135 | 1,100.429 |
| M × L × W | 1,95.085 | 1,96.406 | 1,96.254 | 1,95.655 | 1,95.641 | 1,96.057 | 1,95.611 | 1,95.371 |
| M × L × S | 1,95.798 | 1,97.341 | 1,96.714 | 1,96.013 | 1,96.057 | 1,96.521 | 1,96.284 | 1,95.863 |
| M × D × W | 1,99.916 | 1,103.687 | 1,99.643 | 1,98.698 | 1,99.279 | 1,100.513 | 1,100.873 | 1,100.044 |
| M × D × S | 1,97.354 | 1,99.86 | 1,97.735 | 1,96.871 | 1,97.194 | 1,97.86 | 1,97.928 | 1,97.316 |
| M × W × S | 1,95.356 | 1,96.801 | 1,96.42 | 1,95.786 | 1,95.741 | 1,96.201 | 1,95.871 | 1,95.5 |
| L × D × W | 1,99.575 | 1,103.127 | 1,99.407 | 1,98.483 | 1,98.968 | 1,100.129 | 1,100.445 | 1,99.618 |
| L × D × S | 1,97.347 | 1,100.585 | 1,97.788 | 1,97.011 | 1,97.277 | 1,98.149 | 1,98.149 | 1,97.547 |
| L × W × S | 1,95.504 | 1,96.731 | 1,96.5 | 1,95.827 | 1,95.915 | 1,96.307 | 1,95.961 | 1,95.679 |
| D × W × S | 1,96.175 | 1,98.475 | 1,97.013 | 1,96.336 | 1,96.469 | 1,97.119 | 1,96.857 | 1,96.475 |

SUPPORTING APPENDIX S2.

*Additional plant responses to the treatments*

Across both species, plants grown in drought legacy soils had 15% higher shoot biomass than those in ambient legacy soils (significant main effect). Shoot biomass was higher for well-watered versus droughted plants grown on low diversity soils, but there was no difference in shoot biomass between well-watered and droughted plants grown on high diversity soils (diversity × watering interaction). Shoot biomass of *A. pratensis* was higher when plants were well-watered versus droughted, while the difference between watering treatments for *H. lanatus* was not significant (species × watering interaction). Overall, *H. lanatus* produced more shoot biomass than *A. pratensis*, and well-watered plants produced more shoot biomass than droughted (significant main effects). Root biomass was higher for *H. lanatus* than *A. pratensis*, and well-watered plants grew larger roots (significant main effects). Root to shoot ratios were higher for *A. pratensis* than *H. lanatus* when plants were droughted, but this effect disappeared when plants were well-watered (species × watering interaction). Hyphae and vesicle colonisation was higher in droughted versus well-watered plants and *H. lanatus* had higher arbuscule, hyphae and vesicle colonisation than *A. pratensis* (significant main effects). Shoot %C was affected by maternal origin, diversity legacy and watering interactions, but post-hoc tests revealed that there were no significant differences between the treatment combinations (means not shown). Shoot %C and %N were greater for *A. pratensis* than *H. lanatus*, and shoot %C and %N were higher in droughted than well-watered plants (significant main effects). Shoot C:N ratios were higher in plants grown in ambient legacy than drought legacy soils, for *H. lanatus* than *A. pratensis*, and well-watered than droughted plants; the shoots of *A. pratensis* were less enriched with ^15^N than *H. lanatus* shoots (significant main effects). Also, *A. pratensis* roots had higher %N than *H. lanatus* roots, while *H. lanatus* root C:N ratios were higher than *A. pratensis* (significant main effects). The seeds of *A. pratensis* weighed more than *H. lanatus* seeds (p = 0.039, 0.42 ± 0.08, 0.20 ± 0.02, respectively). Both seed %C and %N were higher in *A. pratensis* than *H. lanatus* (p = 0.003, <0.001, 50.87% ± 1.60, 44.86 ± 0.48 and 5.31 ± 0.51, 2.56 ± 0.09, respectively). Root C concentrations were not affected by any treatments (means not shown).

*Additional soil abiotic and biotic property responses to the treatments*

Microbial C and C:N were both 8% greater in drought legacy than ambient legacy soils (significant main effect). In droughted, drought legacy soils, *H. lanatus* had higher microbial C:N ratios than both *H. lanatus* and *A. pratensis* that were grown in well-watered, ambient legacy soils (drought legacy × watering interaction × species interaction). In drought legacy soils, TIN was higher in droughted versus well-watered pots, but this effect disappeared in ambient legacy soils (drought legacy × watering interaction). Further, in low diversity legacy soils, TIN was higher in droughted than well-watered pots, but this effect disappeared in high diversity legacy soils (diversity legacy × watering interaction). Overall, TIN was higher in drought legacy than ambient legacy soils and soils planted with *A. pratensis* pots had higher TIN concentrations than pots with *H. lanatus* (significant main effects). Overall, URE was 7% higher in drought legacy soils versus ambient legacy soils (significant main effects). Soil GLC was higher in low diversity, drought legacy soils that were well-watered compared to low diversity, drought legacy droughted soils, but this effect disappeared in high diversity soils (drought legacy × diversity legacy × watering interaction). There was a significant drought legacy × watering × species interaction on PER and a significant maternal × drought legacy × watering interaction on POX, but post-hoc tests revealed no differences between treatments (means not shown). URE was higher in *H. lanatus* pots than in *A. pratensis* pots, and URE, GLC, XYL, NAG and PHO were all lower in drought than in well-watered soils (significant main effects). PHO was higher in *A. pratensis* pots. Finally, microbial N was not affected by any of the treatments (Tables S6a,b; means not shown). See Table S8 for details, including means of significant effects not provided in Figures or text.

TABLE S8. Mean ± one standard error of plant (*Alopecurus pratensis* and *Holcus lanatus*) and soil variables that were significantly affected by maternal origin, drought legacy, diversity legacy, watering and/or species that are not shown in figures or mentioned in the text.

|  | Variable | Treatment and mean ± s.e. |
| --- | --- | --- |
| Drought legacy (L) | shoot %N | ambient legacy: 2.30% ± 0.09, drought legacy: 2.54% ± 0.10 |
|  | shoot C:N | ambient legacy: 19.96 ± 0.72, drought legacy: 18.45 ± 0.71 |
|  | microbial C:N | ambient legacy: 4.39 ± 0.08, drought legacy: 4.79 ± 0.14 |
| Watering (W) | shoot biomass | well-watered: 0.33 ± 0.02, droughted: 0.28 ± 0.02 grams |
|  | root biomass | well-watered: 0.29 ± 0.02, droughted: 0.23 ± 0.01 grams |
|  | shoot %C | well-watered: 41.85% ± 0.24, droughted: 42.31% ± 0.16 |
|  | shoot %N | well-watered: 2.36% ± 0.09, droughted: 2.63% ± 0.11 |
|  | shoot C:N | well-watered: 19.31 ± 0.70, droughted: 17.78 ± 0.66 |
|  | hyphae | well-watered: 29.12% ± 1.85, droughted: 36.42% ± 2.15 |
|  | vesicles | well-watered: 6.47% ± 0.56, droughted: 8.91% ± 0.78 |
|  | TIN | well-watered: 6.70 ± 0.62, droughted: 10.09 ± 1.23 μg g^-1^ dw soil |
|  | URE | well-watered: 28.50 ± 0.83, droughted: 26.80 ± 0.74 μg NH_4_-N g^-1^ dw soil |
|  | GLC | well-watered: 357.45 ± 10.25, droughted: 308.06 ± 9.23 nmol h^-1^ g^-1^ dw soil |
|  | XYL | well-watered: 79.50 ± 1.95, droughted: 69.51 ± 2.03 nmol h^-1^ g^-1^ dw soil |
|  | NAG | well-watered: 82.32 ± 2.57, droughted: 68.20 ± 2.27 nmol h^-1^ g^-1^ dw soil |
|  | PHO | well-watered: 1147.19 ± 27.55, droughted: 1033.91 ± 25.28 nmol h^-1^ g^-1^ dw soil |
| Species (S) | shoot biomass | *A. pratensis*: 0.19 ± 0.01, *H. lanatus*: 0.40 ± 0.01 grams |
|  | root biomass | *A. pratensis*: 0.18 ± 0.01, *H. lanatus*: 0.32 ± 0.02 grams |
|  | shoot %C | *A. pratensis*: 43.23% ± 0.13, *H. lanatus*: 41.43% ± 0.20, |
|  | shoot %N | *A. pratensis*: 3.28% ± 0.06, *H. lanatus*: 1.90% ± 0.06 |
|  | shoot ^15^N | *A. pratensis*: 13.31% ± 0.85, *H. lanatus*: 22.32% ± 0.74 |
|  | shoot C:N | *A. pratensis*: 13.45 ± 0.25, *H. lanatus*: 22.50 ± 0.46 |
|  | root %N | *A. pratensis*: 1.03% ± 0.02, *H. lanatus*: 0.89% ± 0.01 |
|  | root C:N | *A. pratensis*: 36.23 ± 0.71, *H. lanatus*: 41.89 ± 0.97 |
|  | arbuscule | *A. pratensis*: 0.92% ± 0.17, *H. lanatus*: 1.2% ± 0.14 |
|  | hyphae | *A. pratensis*: 29.19% ± 2.16, *H. lanatus*: 35.61% ± 1.90 |
|  | vesicles | *A. pratensis*: 6.64% ± 0.83, *H. lanatus*: 8.53% ± 0.57 |
|  | TIN | *A. pratensis*: 12.84 ± 1.39, *H. lanatus*: 5.02 ± 0.30 μg g^-1^ dw soil |
|  | URE | *A. pratensis*: 26.53 ± 0.80, *H. lanatus*: 28.48 ± 0.76 μg NH_4_-N g^-1^ dw soil |
|  | PHO | *A. pratensis*: 1130.16 ± 27.60, *H. lanatus*: 1058.63 ± 26.17 nmol h^-1^ g^-1^ dw soil |
|  | seed weight | *A. pratensis*: 0.42 ± 0.09, *H. lanatus*: 0.19 ± 0.02 mg per seed |
|  | seed %C | *A. pratensis*: 50.87% ± 1.60, *H. lanatus*: 44.87% ± 0.48 |
|  | seed %N | *A. pratensis*: 5.31% ± 0.51, *H. lanatus*: 2.56% ± 0.09 |
| L × Diversity (D) | shoot C:N | high diversity, ambient legacy: 19.32 ± 0.86, drought legacy: 18.96 ± 1.01  low diversity, ambient legacy: 20.57 ± 1.15, drought legacy: 17.92 ± 1.00 |
| L × W | TIN | ambient legacy, well-watered: 6.35 ± 0.64, droughted: 8.58 ± 1.82 μg g^-1^ dw soil  drought legacy, well-watered: 7.22 ± 1.05, droughted: 11.61 ± 1.63 μg g^-1^ dw soil |
| D × W | shoot biomass | high diversity, well-watered: 0.32 ± 0.02, droughted: 0.30 ± 0.02 grams  low diversity, well-watered: 0.35 ± 0.03, droughted: 0.27 ± 0.02 grams |
|  | TIN | high diversity, well-watered: 7.16 ± 0.94, droughted: 8.45 ± 1.01 μg g^-1^ dw soil  low diversity, well-watered: 6.43 ± 0.82, droughted: 11.83 ± 2.27 μg g^-1^ dw soil |
| W × S | shoot biomass | *A. pratensis*: well-watered: 0.22 ± 0.02, droughted: 0.16 ± 0.01 grams  *H. lanatus*: well-watered: 0.42 ± 0.02, droughted: 0.38 ± 0.01 grams |
|  | root to shoot ratio | *A. pratensis*: well-watered: 0.95 ± 0.12, droughted: 1.08 ± 0.10  *H. lanatus*: well-watered: 0.88 ± 0.06, droughted: 0.75 ± 0.04 |
| L × D × W | GLC | high diversity, ambient legacy, well-watered: 363.23 ± 15.78, droughted: 318.09 ± 12.85 nmol h^-1^ g^-1^ dw soil  high diversity, drought legacy, well-watered: 386.30 ± 27.09, droughted: 336.07 ± 22.34 nmol h^-1^ g^-1^ dw soil  low diversity, ambient legacy, well-watered: 322.30 ± 16.73, droughted: 297.98 ± 13.76 nmol h^-1^ g^-1^ dw soil  low diversity, drought legacy, well-watered: 360.37 ± 18.84, droughted: 277.87 ± 21.28 nmol h^-1^ g^-1^ dw soil |
| L × W × S | microbial C:N | ambient legacy, *A. pratensis*: well-watered: 4.24 ± 0.26, droughted: 4.70 ± 0.19  drought legacy, *A. pratensis*: well-watered: 5.02 ± 0.29, droughted: 4.56 ± 0.16  ambient legacy, *H. lanatus*: well-watered: 4.21 ± 0.12, droughted: 4.44 ± 0.12  drought legacy, *H. lanatus*: well-watered: 4.47 ± 0.22, droughted: 5.10 ± 0.36 |

carbon (C), Glucosidase (GLC), N-acetylglucosaminidase (NAG), nitrogen (N), peroxidase (PER), phosphatase (PHO), total inorganic nitrogen (TIN), urease (URE), xylosidase (XYL)

**References**

Brookes, P.C., Landman, A., Pruden, G. & Jenkinson, D.S. (1985). Chloroform fumigation and the release of soil-nitrogen - a rapid direct extraction method to measure microbial biomass nitrogen in soil. *Soil Biology & Biochemistry,* 17**,** 837-842. 10.1016/0038-0717(85)90144-0

DeForest, J.L. (2009). The influence of time, storage temperature, and substrate age on potential soil enzyme activity in acidic forest soils using MUB-linked substrates and L-DOPA. *Soil Biology & Biochemistry,* 41**,** 1180-1186. 10.1016/j.soilbio.2009.02.029

Jackson, C.R., Tyler, H.L. & Millar, J.J. (2013). Determination of microbial extracellular enzyme activity in waters, soils, and sediments using high throughput microplate assays. *Journal of Visualized Experiments***,** 1-9. 10.3791/50399

Kaiser, C., Koranda, M., Kitzler, B., Fuchslueger, L., Schnecker, J., Schweiger, P., . . . Richter, A. (2010). Belowground carbon allocation by trees drives seasonal patterns of extracellular enzyme activities by altering microbial community composition in a beech forest soil. *New Phytologist,* 187**,** 843-858. 10.1111/j.1469-8137.2010.03321.x

Kandeler, E. & Gerber, H. (1988). Short-term assay of soil urease activity using colorimetric determination of ammonium. *Biology and Fertility of Soils,* 6**,** 68-72.

Krom, M.D. (1980). Spectrophotometric determination of ammonia - a study of a modified berthelot reaction using salicylate and dichloroisocyanurate. *Analyst,* 105**,** 305-316. 10.1039/an9800500305
